# Development of a deep learning-based grid walking test for assessing post-stroke motor function in mice

**DOI:** 10.3389/fnbeh.2026.1873231

**Published:** 2026-08-03

**Authors:** Monika Maciag, Vardan T. Karamyan

**Affiliations:** Department of Foundational Medical Studies, William Beaumont School of Medicine, Oakland University, Rochester, MI, United States

**Keywords:** artificial intelligence, automated ladder test, automated motor assessment, behavioral testing, foot fault test, photothrombotic stroke model, stroke recovery, machine learning

## Abstract

Stroke is a leading cause of death and disability, affecting over 15 million people each year. Developing effective therapies depends on accurate, scalable methods to assess sensorimotor recovery in preclinical models. Standard motor tests like grid walking and ladder tests rely on manual scoring, limiting objectivity and throughput. To address this, we compared a commercially available automated ladder test with a newly developed deep learning-based grid walking test using DeepLabCut (DLC) in a photothrombotic stroke model in mice. Our results show that the automated ladder test system failed to detect significant unilateral deficits in stroke animals. It did not capture the expected increase in missteps in the affected forepaw after stroke, even though motor cortex infarcts were confirmed histologically, and the functional impairment was apparent with manual scoring in the grid walking test. In contrast, our DLC-based grid walking model provided fully automated quantification of this widely used test. It achieved millimeter-scale tracking accuracy and detected a ∼3-fold increase in foot faults in stroke-affected mice compared to controls, clearly differentiating between affected and unaffected forepaws for at least 2 weeks post-stroke. These findings expose the limitations of the commercial automated ladder test and demonstrate that the DLC-based pose-estimation-assisted misstep quantification in grid-walk test provides greater sensitivity for detecting motor deficit.

## 1 Introduction

Stroke is a leading cause of disability and death, affecting 15 million people worldwide each year (World Health Organization [WHO], 2025). The majority of these cases, approximately 60%, are ischemic strokes, which occur when a clot obstructs blood flow to the brain (Price et al., 2018; Feigin et al., 2021). To better understand the pathophysiological mechanisms and develop new therapeutic strategies, researchers have established a variety of animal stroke models, with mouse models being the primary focus (Casals et al., 2011; Britsch et al., 2023; Karamyan, 2023; Matur et al., 2023).

One of the most commonly used mouse ischemic stroke models is the photothrombosis model, which induces focal cerebral ischemia by causing endothelial damage, platelet adhesion, and blood clot formation to block cerebral vessels (Li and Zhang, 2021). This model is the least invasive rodent model and is primarily suited for stroke recovery studies (Urbach and Witte, 2016; Inoue et al., 2023). Also, its advantage lies in the flexibility of infarct size and location, allowing targeted ischemic damage to specific brain regions and their associated neurological dysfunctions, thereby reflecting various forms of human ischemia (Li and Zhang, 2021; Nowak et al., 2023). Numerous tests have been developed to assess sensory, motor, and cognitive dysfunctions following stroke injury, and the use of appropriate behavioral tests determines an accurate assessment of stroke outcomes (Balkaya et al., 2013; Ruan and Yao, 2020). Among the most recommended motor tests in stroke recovery studies in mice is the grid walking test, also known as the foot fault test, which has been successfully adapted to evaluate the spontaneous forelimb behavior during gait in the photothrombotic (Clarkson et al., 2013; Alamri et al., 2018; Joy et al., 2019; Al Shoyaib et al., 2021a; van Nieuwenhuijzen et al., 2021), distal (Barios et al., 2016; Syeara et al., 2020) and proximal (Doeppner et al., 2014; Jayaraman et al., 2020; Shi et al., 2021; Li et al., 2022) middle cerebral artery occlusion (MCAO) models of stroke. In this test, mice are allowed to move freely across a grid surface, and motor deficit is quantified based on the number of foot slips made by each paw, allowing for the tracking of both acute and chronic motor impairment following unilateral brain injury (Syeara et al., 2023). Another test based on scoring paw missteps is the horizontal ladder test (aka ladder rung test), where the animal walks across a horizontal ladder with regular or varied spacing between rungs (Balkaya et al., 2013). Especially, in the case of irregular rung conditions, animals cannot anticipate rung locations, and the development of a specific gait pattern is more challenging. As a result, each step demands adjustments in stride length, paw placement, and limb coordination to adapt to the changing rung distances. While healthy animals can quickly adjust to this, the ability is significantly reduced in animals with movement deficits (Metz and Whishaw, 2009).

A significant limitation of grid walking and horizontal ladder tests is the labor-intensive and time-consuming process of manual video analysis. Accurate quantification of motor deficits often requires frame-by-frame review by trained observers, introducing both subjectivity and bottlenecks in data throughput. To overcome these challenges, an Automatic Ladder Test Instrument has been developed (BIOSEB), enabling real-time detection and quantification of paw placement errors across all four limbs during locomotion (Kennedy et al., 2016; Chavez et al., 2019; Zhu et al., 2021; Wang et al., 2024). While this system has shown promise for general motor analysis, its utility in detecting unilateral motor deficits following stroke has not been validated.

Concurrently, the rise of accessible machine learning and deep learning platforms has transformed behavioral neuroscience by enabling user-defined, automated tracking of animal behavior with minimal need for computational expertise. One widely adopted tool in this space is DeepLabCut (DLC; Mathis et al., 2018), an open-source software that allows for markerless pose estimation of user-defined anatomical landmarks using deep learning architectures. DLC has demonstrated broad applicability across experimental paradigms, including social behavior (Zahran et al., 2024), memory processing (Kishi et al., 2025), and models of neurological disease such as spinal cord and traumatic brain injury (Aljovic et al., 2022). Notably, DLC has been successfully used to characterize gait disturbances and misstep frequency in the ladder test following photothrombotic stroke in mice (Weber et al., 2022). On the contrary, despite the widespread use of the grid walking test in assessing post-stroke motor deficit, an automated, high-throughput system for quantifying these kinds of motor impairments remains lacking.

Therefore, this study aimed to evaluate the utility of the commercial BIOSEB automated ladder instrument for assessing unilateral forepaw dysfunction in the mouse model of photothrombotic stroke. In parallel, we developed and validated a DLC-based scoring model for the grid walking test, providing an artificial intelligence (AI)-powered method for objectively quantifying missed steps according to the traditional scoring criteria of this widely used motor test. Our analysis demonstrates that the automated ladder instrument lacks sufficient sensitivity to reliably detect unilateral motor deficits following photothrombotic stroke in mice. In contrast, our newly developed the DLC-based scoring method consistently documented motor impairment in the grid walking test over 2 weeks after stroke, highlighting its superior utility for longitudinal assessment of motor dysfunction.

## 2 Materials and methods

### 2.1 Animals and study design

**FIGURE 1:**
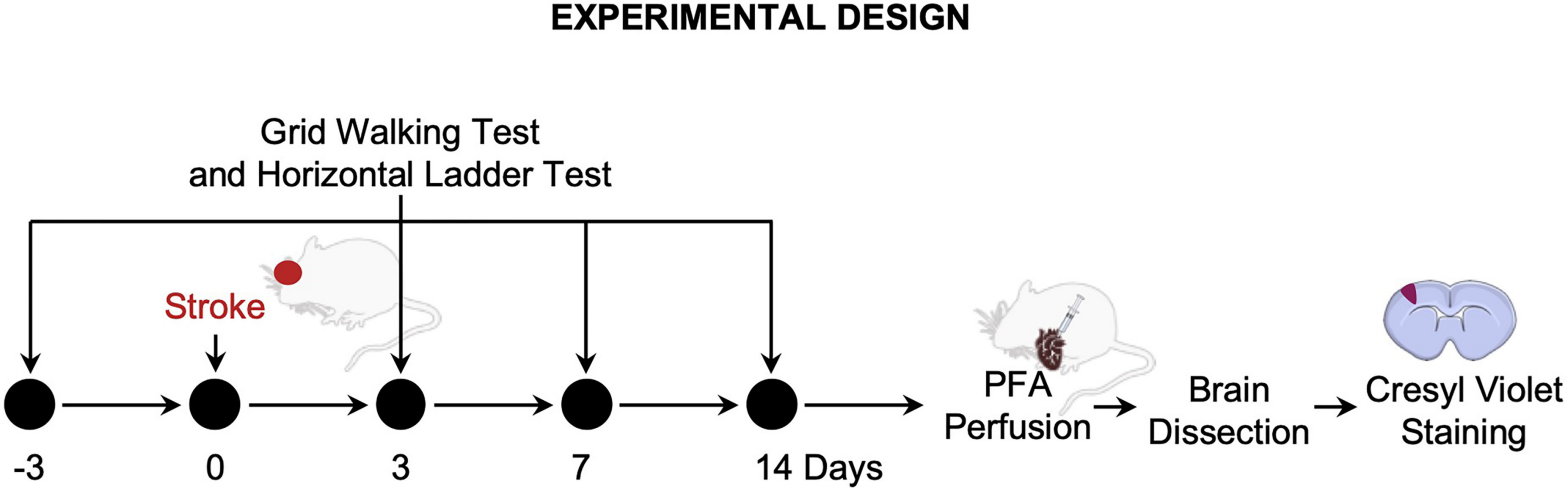
Experimental framework. After the baseline evaluation of motor function (day-3), mice were randomly assigned to sham and stroke groups. Stroke was induced by photothrombosis (day 0). Motor function was assessed on post-stroke days 3, 7, and 14 (PSD3, PSD7, PSD14) using the grid walking and automated horizontal ladder tests. On PSD14, mice were euthanized, perfused with 4% paraformaldehyde (PFA), and their brains were removed and processed for sectioning. Cresyl violet staining was then used to evaluate the location and volume of infarction.

A group of twenty 8–10-week-old male CD-1 mice was received from Charles River Laboratories and maintained at the animal facility at Oakland University, USA. The mice were housed under standard laboratory conditions with a 12-h light/dark cycle and fed *ad libitum* in groups of 2–4 per cage. All mice were allowed to acclimate to the experimental room for 1 week before the start of the experimental procedure and 30 min before each test. To minimize handling stress, mice were individually handled by two investigators for ∼2 min twice daily for at least 5 days before baseline assessment. On the day of testing, the mice weighed between 37 g and 47 g. Before surgeries, mice were randomly assigned to the sham or stroke group using an online randomization generator (Randomizer, 2024). Group allocations were prepared by an investigator not involved in behavioral data analysis and were disclosed only after completion of manual misstep scoring to ensure allocation concealment and prevent observer bias. The behavioral cohort comprised ten animals per group. No *a priori* power calculation was performed; however, based on our previous studies (Syeara et al., 2020; Al Shoyaib et al., 2021a) and a detailed analysis of the obtained data from manual scoring, the variability of the stroke-affected forepaw across all testing days was 23% (SD/mean × 100). Using this variability, a *post hoc* power analysis indicated that four animals per group would be required to detect a 50% difference between two groups with 80% power at a two-sided α-level of 0.05 (see Supplementary Table 1). Three days after baseline evaluation of the motor function, the animals underwent photothrombosis (Figure 1). After the stroke, mice were housed individually, and no animal died or was eliminated from the study. Motor function was assessed on post-stroke days 3, 7, and 14 (PSD3, PSD7, and PSD14), first undergoing the grid walking test and followed up by the automated horizontal ladder test. This order was selected because the grid walking task does not involve repetitive or learned movements that could influence subsequent ladder performance. Additionally, both tests were separated by sufficient rest intervals for each animal (30–60 min). The investigator conducting the motor tests was blinded to the experimental group identity. For histological analyses, six brains per group were processed, reasoned by a low inter-individual variability of the infarction and by practical constraints in histological throughput. Animals selected for histology were chosen *a priori*. The study was approved by the Oakland University Institutional Animal Care and Use Committee (protocol # 2023-1208). All animal studies complied with the ARRIVE guidelines and were conducted in accordance with the policies and laws of the NIH Office of Laboratory Animal Welfare.

### 2.2 Photothrombotic stroke model

Photothrombosis was induced according to our previously published protocol (Al Shoyaib et al., 2021b; Alamri et al., 2023). In brief, the right hemisphere (1.5 mm lateral from Bregma 0) was illuminated through the intact skull using cold light from a fiber optic illuminator (2-mm diameter) light source for 15 min. The light irradiation was started 5 min after intraperitoneal injection of Rose Bengal solution (8 mg/ml, 10 ml/kg volume, MilliporeSigma, USA) to mice under isoflurane (Covetrus, USA) anesthesia at 36.9 ± 0.5 °C. Immediately following surgery, animals were placed in individual recovery cages with controlled heating (36.9 ± 0.5 °C) for approximately 70 min before returning to a new home cage. The new home cage contained a nesting box and paper strips, in addition to water and food. For sham animals, the same procedure was followed, except that there was no light illumination. Mice were monitored at least twice daily for signs of pain, distress, or seizures. Humane endpoints included body weight loss exceeding 15%, immobility, lack of grooming, or inability to access food or water. Additional exclusion criterion included death during surgery (<5%). No animals reached humane endpoints or were excluded from the study. Throughout the text, the left forepaw of both sham and stroke mice is referred to as the “affected forepaw,” whereas the right forepaw is referred to as the “unaffected forepaw.”

### 2.3 Grid walking test

In the grid walking test, mice were allowed to walk freely on an elevated wire grid with a total area of 33 cm × 20 cm and a 1.2 cm square wire mesh, as described in our earlier publications (Alamri et al., 2021; Syeara et al., 2023). The test took place in a well-lit experimental room between 8 AM and 11 AM during the light cycle for animals. Each mouse was placed individually on the grid, and their movements were recorded on video for 5 min using a full HD action camera equipped with a 12-megapixel wide-angle lens, capturing video at 30 frames per second (Sports HD DV, model XPC-A101-BLACK; Supplementary Videos 1, 2). The distance between the front edge of the grid and the camera lens was 18.5 cm. The test arena was thoroughly cleaned with 20% isopropanol between each trial. The recordings were later analyzed using Behavioral Observation Research Interactive Software (BORIS, software version: 8.25.4) (Friard and Gamba, 2016) to determine the number of affected forepaw and unaffected forepaw missteps, defined as slipping or being placed within the grid hole, as well as the total number of steps. Manual scoring of the grid walking test was performed by a single trained investigator who was blinded to experimental group allocation. The percentage of each forepaw misstep over the total steps was then calculated as a primary endpoint of motor dysfunction. Also, the number of steps taken per second was analyzed.

### 2.4 Automatic ladder test

The horizontal ladder test was carried out using the Automatic Ladder from BIOSEB *In Vivo* Research Instruments Co., [France; product: BIO-LADDERTEST (Automatic Ladder Test [BIOSEB], 2025)]. The mouse corridor was 120 cm long and had two side walls spaced 6 cm apart. Both regular (0.5 cm) and irregular (up to 2.5 cm) ladder prong patterns were used on a random basis. The ladder was set at the lowest possible height to accurately detect the animal’s body position and missteps, using two rows of infrared beams positioned above and below the ladder rungs. The test was conducted in the same experimental room after completing the testing on the grid walk. The mouse was placed at the beginning of the corridor immediately after starting the tracking by the BIO-LADDERSOFT software (software version: 1.4.4). In the software, the minimum and maximum average run speed thresholds were set to 1.0 cm/s and 100.0 cm/s, respectively. The instantaneous speed mobility threshold, which determines the filtered run duration by excluding periods when the animal’s speed falls below the defined cutoff, was set to 0.1 cm/s. One day before the baseline assessment, each mouse underwent five “pre-runs” on the corridor to acclimate to the ladder apparatus. A full walk through the corridor without changing directions was considered a successful trial, and it was facilitated by placing the home cage of the animal at the end of the walking corridor. Up to 15 runs were completed for each mouse per experimental day. The acquired data were analyzed for both the first and last 5 runs, as well as for all 15 runs separately. The test arena was thoroughly cleaned with 20% isopropanol between animals. The BIO-LADDERSOFT software (software version: 1.4.4) from the BIOSEB was used to calculate the number of affected forepaw and unaffected forepaw missteps, defined as slipping or being placed between bars, as well as the total steps. The percentage of each forepaw misstep over the total steps was calculated as the primary endpoint of motor dysfunction. Additionally, to assess locomotion and gait function, the time taken to run through the corridor was measured and expressed as the number of steps per second.

### 2.5 DeepLabCut (DLC) scoring for the grid walking test

Our next aim was to develop a deep learning-based model capable of quantifying the number of missteps and total steps performed by mice during locomotion in the grid walking test. To achieve this, we used DLC (version 3.0.0rc6, installed via Anaconda environment), an open-source computer vision toolbox designed for automatic, markerless tracking of user-defined anatomical features (Mathis et al., 2018). Video recordings of the grid walking test were used to create the model. Frames containing visible missteps were manually selected and included in the training phase. To ensure comprehensive representation of missteps across different perspectives and depths, annotated frames were intentionally selected from multiple camera viewpoints, including back, front, right, and left positions relative to the grid, as well as from varying distances of the animal to the camera recorded from a lateral view on different experimental days. To improve model efficiency, videos were preprocessed by cropping to remove background elements, which helped accelerate the training process. The final image resolution was 1918 × 614 pixels. The neural network backbone was based on ResNet-50, and training was performed on a total of 4,065 manually labeled frames extracted from videos using k-means clustering. To reduce potential data leakage between training and test sets, missteps were selected from 10 mice (half of the animals used in this study) covering multiple perspectives and angles, with 95% of training frames kept independent of the test set. An experienced annotator manually labeled eight anatomical joints: nose, right ear, left ear, right shoulder, left shoulder, right hip, left hip, and the base of the tail. In addition, four types of missteps were annotated when present: right front, left front, right rear, and left rear missteps. Missteps were defined consistently with the criteria used in manual scoring, detailed above. Due to a greater frequency of left front paw missteps compared to the right, approximately three-fourths of the labeled frames included left front missteps, with the remaining frames focusing on the right front paw. The labeled dataset was randomly split into training (95%) and test (5%) sets, and the network was trained for 1,030,000 iterations. The network was trained and evaluated across five data shuffles, with the number of annotated frames progressively increasing. Final video analysis was performed using the model trained on the fifth shuffle. Labeling accuracy was assessed using the root-mean-square error (RMSE) in pixel units (later converted to centimeters), calculated separately for the training and test sets. Kinematic analysis was performed using the extracted x- and y-coordinate data.

Right and left front misstep analysis: The number of right and left front paw missteps was quantified based on the predicted likelihood scores for the labeled events “right front misstep” and “left front misstep.” Because the model was trained on approximately three times more frames depicting left (affected) than right (unaffected) forepaw missteps, we applied different likelihood thresholds to balance detection sensitivity: 0.7 for left (affected) and 0.2 for right (unaffected) forepaw missteps. The likelihood thresholds were established based on preliminary calibration. An additional analysis was performed using a threshold of 0.7 for the right forepaw. The resulting data were further refined by consolidating consecutive detections; if a misstep was identified in two subsequent frames, it was counted as a single event to avoid overestimation due to frame-by-frame continuity. The percentage of each forepaw misstep over the total steps was then calculated as an indicator of motor dysfunction.

Total step analysis: The number of steps performed by the right and left forelimbs was calculated based on the labeled positions of the right and left shoulders. Step speed was defined as the diagonal distance traveled between two consecutive frames. Distances in pixel units (1 cm = 70 pixels) were converted to centimeters, and time was calculated by converting frames to seconds (30 frames = 1 s). To detect and correct potential tracking artifacts, step values were excluded if the displacement between two consecutive frames exceeded three standard deviations (SD) of the overall placement distance. To identify individual steps during each run, we classified movement phases into step and swing periods. A step was defined if the limb’s speed exceeded 1 cm/s at baseline and 5 cm/s on subsequent test days, for both sham and stroke groups. The difference in speed thresholds between baseline and later days likely reflects the increased exploratory behavior typically observed in mice when first exposed to a novel environment (Pisula and Siegel, 2005). Based on this, the number of steps per second was calculated.

### 2.6 Cryosectioning

After completing the last set of behavioral tests on PSD14, the mice were euthanized by deep isoflurane anesthesia followed by cardiac perfusion of 4% paraformaldehyde (PFA, Thermo Scientific, USA) diluted in phosphate-buffered saline (PBS). The whole brain was removed and incubated in 4% PFA overnight, followed by cryopreservation in 20% and 30% sucrose solution in PBS at 4 °C. The frozen mouse brains were sectioned using a cryostat (Leica CM3050 S) with the cryochamber and object temperatures set at −20 °C. The coronal brain sections (50 μm) were stored in a cryoprotective solution at −20°C until further use.

### 2.7 Cresyl violet staining and infarct size assessment

PFA-fixed brain sections from both sham and stroke groups were mounted on Superfrost Plus microscope slides (Fisher Scientific, USA) and stained with cresyl violet (Thermo Scientific, USA) according to our established protocol. The slides were immersed in 100% ethanol and xylene for 1 h. Then, they were slowly rehydrated in decreasing concentrations of ethanol (100% ethanol and 95% ethanol for 4 min, 70% ethanol for 2 min) before being placed in distilled water for 4 min. Once rehydrated, the slides were stained with 0.1% cresyl violet solution for 5 min and then placed in distilled water for 2 min. Following this step, the slides were slowly dehydrated in increasing ethanol concentrations (70% ethanol for 2 min, 95% ethanol for 4 min, and 100% ethanol for 10 min) before being immersed in xylene for 9 min. Finally, the slides were removed from xylene and mounted with a Permount Mounting Medium (Fisher Chemical, USA). Infarct area was quantified using ImageJ software (version: 1.54f, National Institutes of Health, Bethesda, MD, USA), and it was used to calculate the area and volume of infarction. As a secondary endpoint assessing the performance of the model, the relationship between infarct volume and infarct location and the misstep ratio on PSD14 was evaluated using Pearson correlation; if normality assumptions were violated, Spearman’s rank correlation was applied instead.

### 2.8 Statistical analysis

Statistical analyses were conducted using mixed-effects models with restricted maximum likelihood (REML) estimation, applying Greenhouse-Geisser correction for repeated measures. Time (PSD) and group (sham *vs*. stroke) were included as fixed effects, with their interaction, and subject (mouse) as a random effect. Tukey’s multiple comparisons test was applied to control the familywise error rate (FWER) across groups and time points, with individual variances computed per comparison. Data were assessed for normality (Shapiro-Wilk test) and homogeneity of variances. Partial eta squared (η^2^_*p*_) was computed from F-statistics to quantify effect sizes, with 95% confidence intervals (CI) obtained using MBESS-based methods and non-central F-distribution methods for effects with non-integer degrees of freedom. *P*-values < 0.05 were considered statistically significant. Data are presented as mean ± the standard error of the mean (SEM).

To assess the predictive value of the DLC model, additional secondary endpoints were included to compare manual and DLC-based scoring, consisting of Bland-Altman analysis, intraclass correlation coefficients (ICC), correlation analyses, and receiver operating characteristic (ROC) and precision–recall (PR) analyses: Agreement between manual and DLC-based misstep scoring was assessed using Bland-Altman analysis and ICC. The Bland-Altman plot was used to quantify systematic bias and limits of agreement between methods. Bland-Altman differences were calculated as manual scoring minus DLC scoring. Reliability was evaluated using a two-way mixed-effects, single-rater, absolute-agreement ICC model. A histogram was generated using the difference in the percentage of missed steps calculated as Manual score - DLC score for each individual observation. The correlation between manual and DLC-based misstep scores was assessed using the Spearman correlation. ROC and precision-recall PR curves were generated to evaluate the discriminative performance of the DLC-based scoring. An *a priori* threshold was defined based on the distribution of the sham group, calculated as the mean plus two standard deviations (11.56%). Mice with values ≤11.56% were classified as normal, whereas those with values >11.56% were classified as impaired. Manual scores were binarized according to this predefined threshold for subsequent classification analyses. The optimal DLC classification threshold was determined using ROC curve analysis and the Youden index (sensitivity + specificity - 1). For PR analysis, 33 of 160 trials (21%) were classified as misstep-positive and 127 of 160 trials (79%) as negative, corresponding to a baseline precision of 0.206. All analyses were performed using the percentage of missteps for both affected (left) and unaffected (right) forepaws, pooled across all testing days (baseline and three post-operative days), derived from 20 animals (10 per experimental group: sham and stroke), resulting in a total of 160 matched data points (20 animals × 4 days × 2 limbs). All analyses were performed using Prism v10.1.1 from GraphPad software or RStudio (Version 2025.09.1+401).

## 3 Results

### 3.1 The grid walking test detects a severe motor deficit in the affected forepaw

**FIGURE 2:**
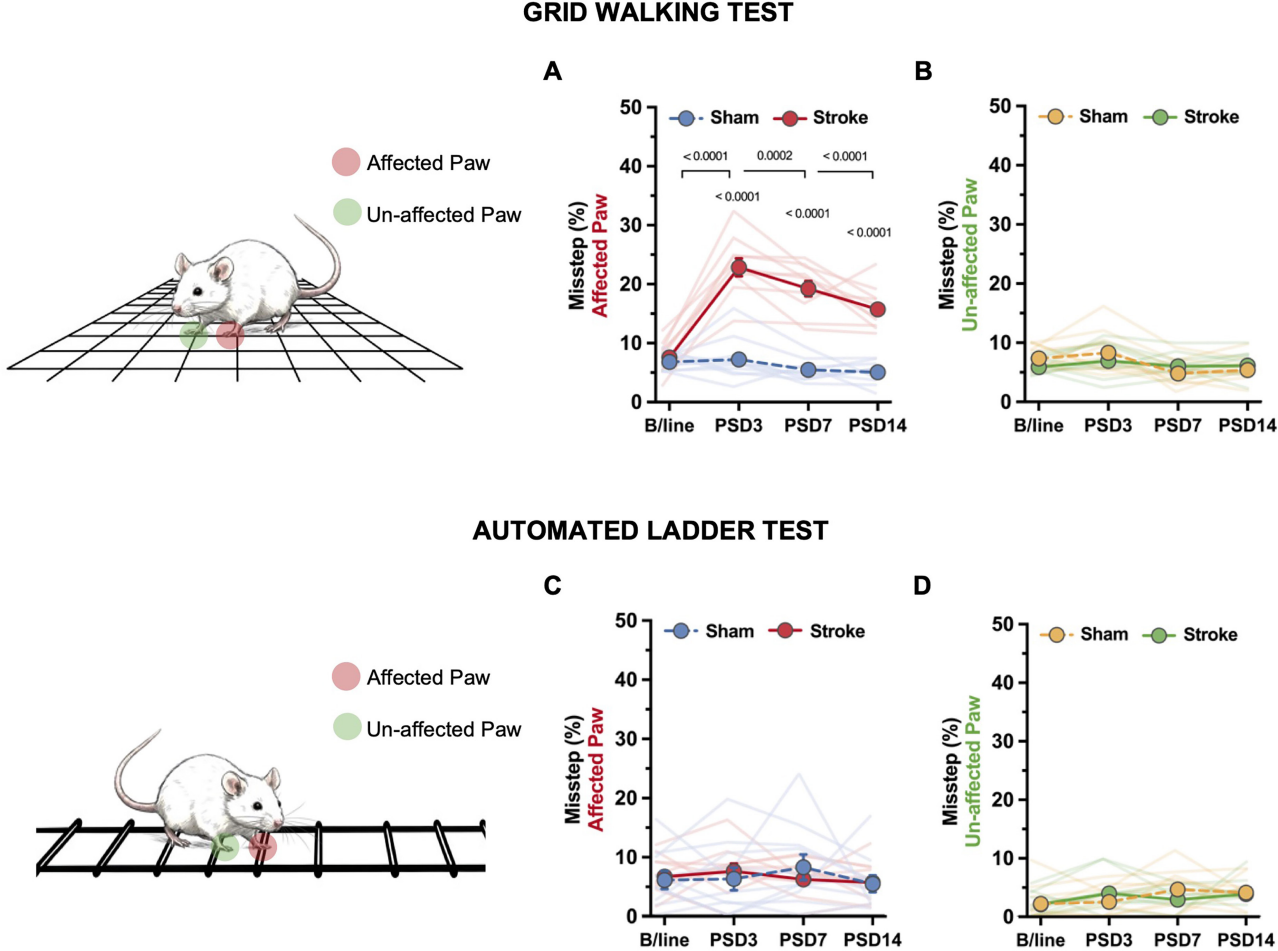
Grid walking, but not automated ladder test, reveals persistent motor deficit in mice after stroke. **(A)** The grid walking test revealed a significant and sustained deficit in the motor function of the affected front paw, with the maximum effect on post-stroke day 3 (PSD3), showing 22.83% missteps. Although the deficit decreased over time, it remained statistically significant on post-stroke days 7 (PSD7) and 14 (PSD14). No differences were observed in sham mice across days or between sham and stroke groups at baseline assessment. **(B)** No apparent motor deficit was observed in the unaffected front paw, i.e., no changes in missteps in the grid walking test. **(C)** The commercial automated ladder instrument (BIOSEB) did not detect significant changes in missteps for the affected front paw in stroke mice on PSD3, PSD7, or PSD14. **(D)** Likewise, the unaffected front paw missteps measured in the automated ladder test did not change significantly during the same period. Data were analyzed using repeated measures mixed-effects model followed by Tukey’s post-hoc test. *P*-values < 0.05 were considered statistically significant. The bold, solid and dashed lines represent the mean data ± standard error of the mean (SEM), *n* = 10/group. The faint lines show the data for individual animals in each experimental group.

First, we evaluated contralateral (affected, i.e., left) forepaw performance in the grid walking test as the primary endpoint, and as expected, we documented that photothrombotic stroke resulted in a significant and sustained increase in misstep percentage. Manual, frame-by-frame evaluation of video recordings revealed a statistically significant day × group interaction for the affected front paw (repeated measures mixed-effects model: within experimental groups: *F* (1, 18) = 102.4, *p* < 0.0001; within experimental days: *F* (2.375, 42.76) = 31.95, *p* < 0.0001; interaction: *F* (3, 54) = 31.84, *p* < 0.0001; Figure 2A). In the stroke group, the maximum effect was achieved on PSD3, with 22.83% of the affected forepaw missteps and an absolute mean difference between baseline and PSD3 of 15.28 percentage points (*p* < 0.0001, 95% CI: 11.12–19.44). Over time, the effect decreased but remained statistically significant. The mean difference of the affected front paw missteps was 11.67 percentage points between baseline and PSD7 (*p* = 0.0002, 95% CI: 6.73–16.61) and 8.19 percentage points between baseline and PSD14 (*p* < 0.0001, 95% CI: 5.02–11.35). There was no difference in the sham mice between days. Within-day comparisons of experimental groups showed no difference between sham and stroke groups at baseline (+0.76 pp, *p* = 0.4365, 95% CI: −1.27 to 2.78) but a statistically significant difference at PSD3 (+15.59 pp, *p* < 0.0001, 95% CI: 11.54–19.63), PSD7 (+13.76 pp, *p* < 0.0001, 95% CI: 10.66–16.85), and PSD14 (+10.68 pp, *p* < 0.0001, 95% CI: 7.98–13.38). We did not observe a substantial difference in the number of missteps for the unaffected front paw over time within the stroke group or between sham and stroke-affected mice on the same day (Figure 2B). Collectively, these results confirm sustained motor deficit in mice for up to 14 days after photothrombotic stroke. Data for *F*-values, partial η^2^ with 95% confidence intervals, and Cohen’s f for all effects are provided in Supplementary Table 2.

### 3.2 The automatic ladder instrument does not detect motor deficit in the affected forepaw

The motor function of the same experimental animals was also evaluated in the commercial automated ladder test using the BIO-LADDERTEST instrument (BIOSEB, France). Contrary to our expectation, we did not observe significant day x group interaction when comparing the affected front paw missteps (primary endpoint, repeated measures mixed-effects model: within experimental groups: *F* (1, 18) = 7.10 × 10^–7^, *p* = 0.9993; within experimental days: *F* (2.461, 44.31) = 0.7337, *p* = 0.5123; interaction: *F* (3, 54) = 0.7297, *p* = 0.5388; Figure 2C). *Post hoc* comparisons within the stroke group, relative to baseline, also failed to show significant changes in the misstep rate: PSD3 (+0.91 pp, *p* = 0.9019, 95% CI: −3.28 to 5.11), PSD7 (–0.46 pp, *p* = 0.9730, 95% CI: −3.83 to 2.92), and PSD14 (–0.98 pp, *p* = 0.9174, 95% CI: −5.75 to 3.80). Similarly, comparisons between the sham and stroke groups on individual test days showed no statistically significant differences (baseline: +0.57 pp, *p* = 0.7518, 95% CI: −3.21 to 4.36; PSD3: +1.29 pp, *p* = 0.5872, 95% CI: −3.65 to 6.23; PSD7: −2.07 pp, *p* = 0.4056, 95% CI: −7.29 to 3.15; PSD14: +0.21 pp, *p* = 0.9051, 95% CI: −3.51 to 3.93). For the unaffected front paw, there was no difference within either sham or stroke-affected groups or between sham and stroke mice over time (Figure 2D). Since these data were calculated based on all 15 runs on the ladder for each experimental animal, we also examined whether motor deficit may be detectable when analyzing the first 5 runs. In this case also, no differences were observed between the sham and stroke groups for either the affected or unaffected forepaw (for the affected front paw: repeated measures mixed-effects model: within experimental groups: *F* (1, 18) = 0.2076, *p* = 0.6541; within experimental days: *F* (2.606, 46.92) = 2.300, *p* = 0.0975; interaction: *F* (3, 54) = 1.277, *p* = 0.2916; see Supplementary Figures 1A,B). Also, no significant differences were detected when analyzing only the last five runs (for the affected front paw: repeated measures mixed-effects model: within experimental groups: *F* (1, 18) = 0.2490, *p* = 0.6238; within experimental days: *F* (2.376, 42.76) = 2.833, *p* = 0.0612; interaction: *F* (3, 54) = 0.5563, *p* = 0.6462; Supplementary Figures 1C,D).

Given that the ladder test captures both total steps and missteps, and our outcomes are expressed as the percentage of missteps relative to total steps, we sought to determine whether the lack of observable post-stroke motor deficits might be due to inaccuracies in step counting. Quantitative analysis of left and right forelimb steps revealed no significant differences between affected and unaffected forelimbs (Supplementary Figures 1E,F), nor between the sham and stroke groups, with animals consistently performing approximately two steps per second (Supplementary Figures 1G, H).

### 3.3 Deep learning-based assessment of missteps in the grid walking test

**FIGURE 3:**
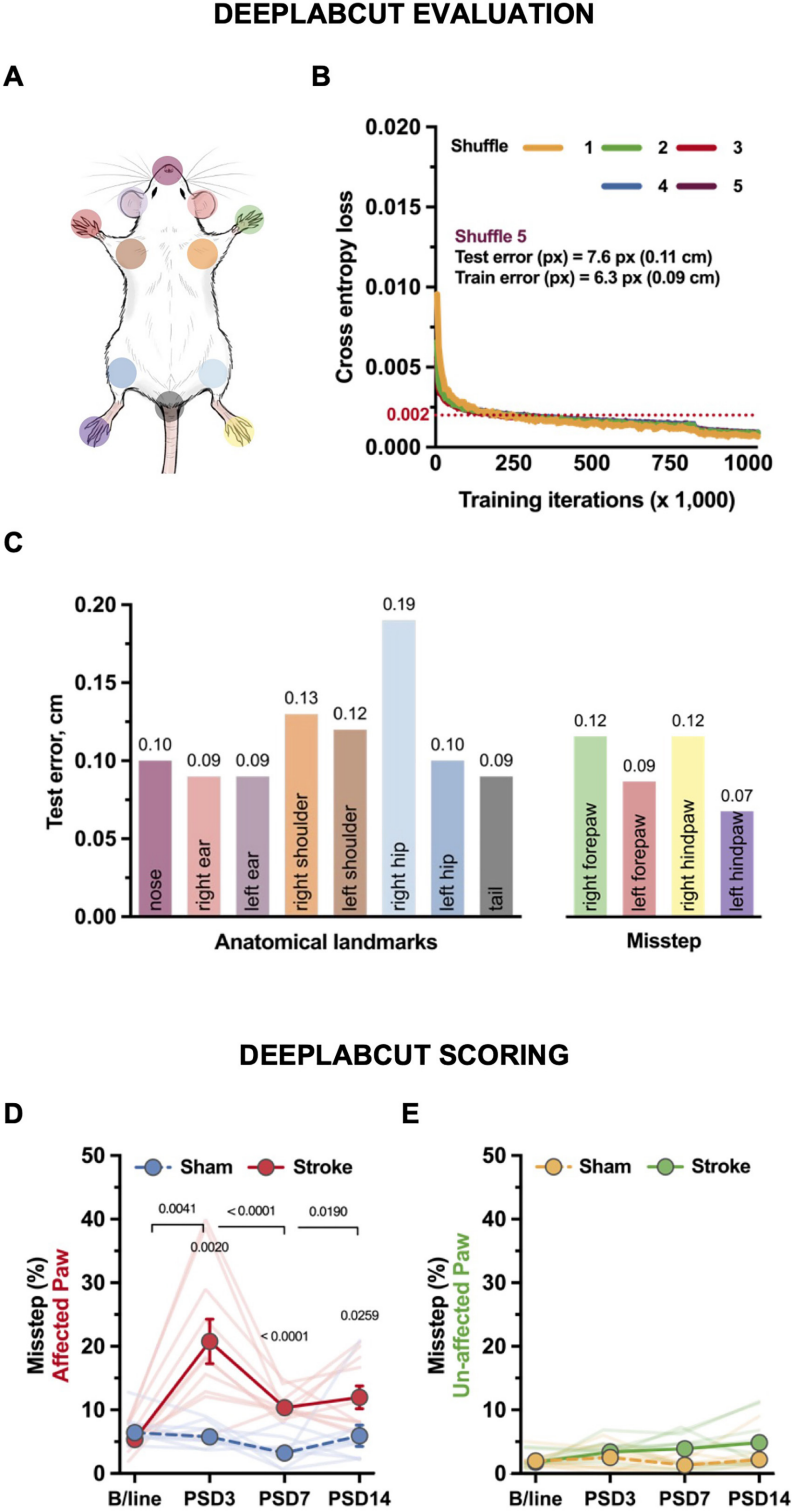
DeepLabCut (DLC)-based analysis revealed significant motor dysfunction in the post-stroke mice. **(A)** Key anatomical landmarks, including the nose, left and right ears, shoulders, hips, and tail base, and four distinct types of missteps were labeled: right forepaw, left forepaw, right hindpaw, and left hindpaw. **(B)** The DLC network achieved a cross-entropy loss below 0.002 and plateaued after 500,000 training iterations. **(C)** Tracking accuracy across landmarks in the test set ranged from 0.07 to 0.19 cm. **(D)** DLC-based scoring mirrored the results of manual scoring in the grid walking test, revealing a significant motor deficit in the affected front paw on post-stroke days 3 (PSD3), 7 (PSD7), and 14 (PSD14). No significant changes were observed in sham-operated mice or between sham and stroke groups at baseline. **(E)** No increase in missteps was observed for the unaffected front paw. Data were analyzed using a repeated measures mixed-effects model followed by Tukey’s post-hoc test. *P*-values < 0.05 were considered statistically significant. The bold, solid and dashed lines represent the mean data ± standard error of the mean (SEM), *n* = 10/group. The faint lines show the data for individual animals in each experimental group.

To establish a automated approach for quantifying functional motor recovery in mice following stroke, we applied the grid walking test in combination with the open-source deep learning software DLC. DLC neural networks were trained using a ResNet-50 backbone by manually annotating 4,065 frames selected at random from videos of multiple mice. Anatomical landmarks were defined following established guidelines to enable comprehensive analysis of locomotor coordination, posture, and inter-limb relationships (Preisig et al., 2016; Mathis et al., 2018; Sheppard et al., 2022; Weber et al., 2022). Eight key body parts were labeled: nose, left ear, right ear, left shoulder, right shoulder, left hip, right hip, and tail base. Additionally, four distinct types of missteps – right forepaw, left forepaw, right hindpaw, and left hindpaw – were annotated where present (Figure 3A). The network achieved a cross-entropy loss plateauing below 0.002, typically at 500,000 training iterations (Figure 3B). This was viewed that the model reached a stable performance level, and there was a minimal divergence between predicted and true labels. Tracking accuracy, evaluated using RMSE, reached 7.6 pixels (≈0.11 cm) on the test set and 6.3 pixels (≈0.09 cm) on the train set, without using any *p*-cutoff value. Per-body-part RMSEs in the test set ranged from 0.09 to 0.19 cm across anatomical landmarks and between 0.07 and 0.12 cm across misstep labeling, reflecting consistent sub-centimeter accuracy (Figure 3C, Supplementary Figure 2A and Supplementary Table 3).

The trained model was subsequently applied to the recorded videos of the grid walking test to extract the coordinates of each labeled body part and to detect missteps in both the affected and unaffected forepaws. The DLC-based model generated data showing a significant and sustained deficit in the function of the affected forepaw, evidenced by an increased percentage of missteps (primary endpoint, repeated measures mixed-effects model: within experimental groups: *F* (1, 18) = 22.22, *p* = 0.0002; within experimental days: *F* (2.150, 38.70) = 10.46, *p* = 0.0002; interaction: *F* (3, 54) = 10.43, *p* < 0.0001; Figure 3D), consistent with our results obtained through manual scoring presented in Figure 2. In the stroke group, the maximum effect was achieved on PSD3, with 20.76% of the affected front paw missteps and an absolute mean difference between baseline and PSD3 of 15.39 percentage points (*p* = 0.0041, 95% CI: 5.49–25.29), 4.98 percentage points between baseline and PSD7 (*p* < 0.0001, 95% CI: 3.13–6.83) and 6.60 percentage points between baseline and PSD14 (*p* = 0.0190, 95% CI: 1.13–12.06). There was no difference in the sham mice between days. Similarly, within-day comparisons of experimental groups showed no difference between sham and stroke groups at baseline (–1.07 pp, *p* = 0.2969, 95% CI: −3.17 to 1.03) but a statistically significant difference on PSD3 (+14.97 pp, #*p* = 0.0020, 95% CI: 7.01–22.93), PSD7 (+7.12 pp, *p* < 0.0001, 95% CI: 5.08–9.16), and PSD14 (+6.02 pp, *p* = 0.0259, 95% CI: 0.81–11.22). Missteps of the unaffected paw did not differ significantly between groups (Figure 3E). As demonstrated in Supplementary Figure 2B, using a uniform 0.7 threshold across paws artificially suppresses misstep detection on the unaffected side, keeping values at zero. Using the coordinates of the right and left shoulders, the number of steps taken was calculated and compared to manually scored data. The results showed nearly identical step counts, with no significant differences between manual and DLC-based scoring in both the sham (Supplementary Figure 2C) and stroke groups (Supplementary Figure 2D), with animals performing approximately one step per second. Together, these findings confirm that the DLC-based model reliably detects sustained motor deficits in mice for up to 14 days following photothrombotic stroke in the grid walking test.

### 3.4 The infarction involved the primary motor area across all stroke-affected mice

**FIGURE 4:**
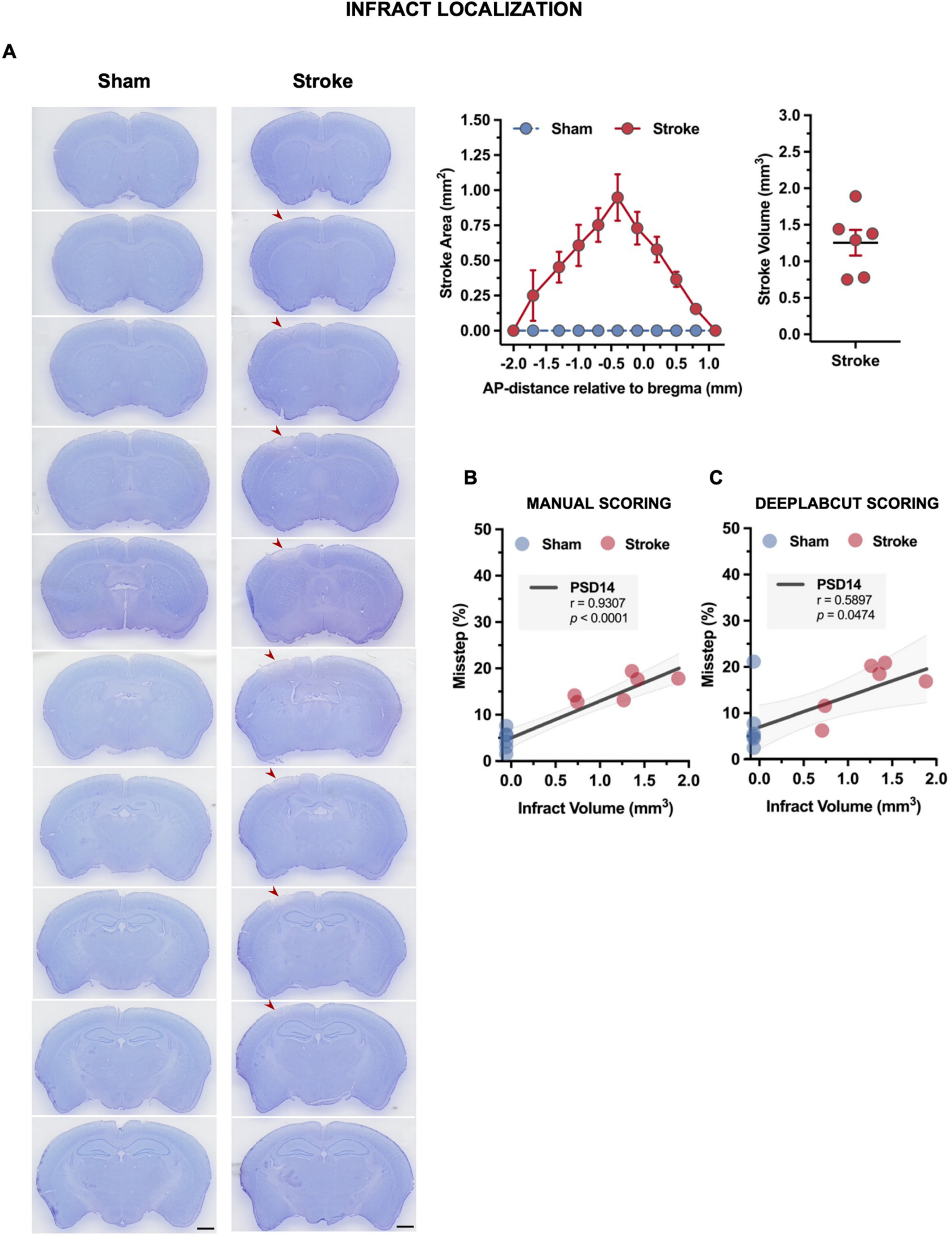
The infarction in the cerebral cortex involves the primary motor area in the right hemisphere of stroke-affected mice. Representative cresyl violet-stained images of sham (left) and stroke (right) mouse brain sections on post-stroke day 14 (PSD14) are depicted. **(A)** Arrows indicate the location of the infarction in the primary motor cortex, with the largest affected area measuring 0.95 ± 0.16 mm^2^ and an infarct volume of 1.25 ± 0.18 mm^3^. Infarct volume showed a positive correlation with motor impairment of the affected forepaw, as measured by both manual **(B)** and DLC-based misstep scoring **(C)**. Lesion position was likewise predictive of motor outcome, with more rostrally located infarcts associated with fewer missteps. Data were analyzed using Pearson **(B)** and Spearman **(C)** correlations. *P*-values <0.05 were considered statistically significant. Data are shown as a mean ± standard error of the mean (SEM), *n = 6*/group. Scale bar: 1 mm.

To confirm the stroke, we evaluated the location and volume of cerebral infarction on PSD14 in mice. PFA-fixed brain sections from all experimental groups were stained with cresyl violet and examined histologically. The infarction of the cerebral cortex involves the primary motor area in the right hemisphere, with its peak located ∼0.4 mm posterior to bregma and a mean affected area of 0.95 ± 0.16 mm^2^ across stroke-affected animals. Volumetric analysis revealed that the mean infarct volume was 1.25 ± 0.18 mm^3^ in stroke-affected mice, with no observable infarction in the sham group (Figure 4A).

As both manual and DLC-based scoring met the primary endpoint criterion (i.e., a significant increase in misstep percentage in the stroke group), we conducted a secondary analysis to assess the relationship between infarct size and location, and the misstep ratio. We observed a strong association between infarct volume and motor impairment of the affected forepaw. Infarct size correlated with the number of missteps detected by manual scoring (*r* = 0.93, 95% CI: 0.76–0.98, *p* < 0.0001; Figure 4B) and also showed a significant, albeit weaker, correlation using DLC-based scoring (*r* = 0.59, 95% CI: 0.005–0.87, *p* = 0.047; Figure 4C).

Also, the rostro-caudal position of the lesion was related to motor performance. Infarcts with a center of mass located more rostrally (closer to bregma) were associated with fewer missteps in the manual dataset (*r* = −0.8641, 95% CI: −0.98 to −0.18, *p* = 0.0265; Supplementary Figure 3A). The DLC-based analysis revealed a similar directional trend (*r* = −0.5419, 95% CI: −0.94 to 0.47), although it did not reach statistical significance (*p* = 0.2592; Supplementary Figure 3B).

### 3.5 Agreement, correlation, and misstep detection performance

**FIGURE 5:**
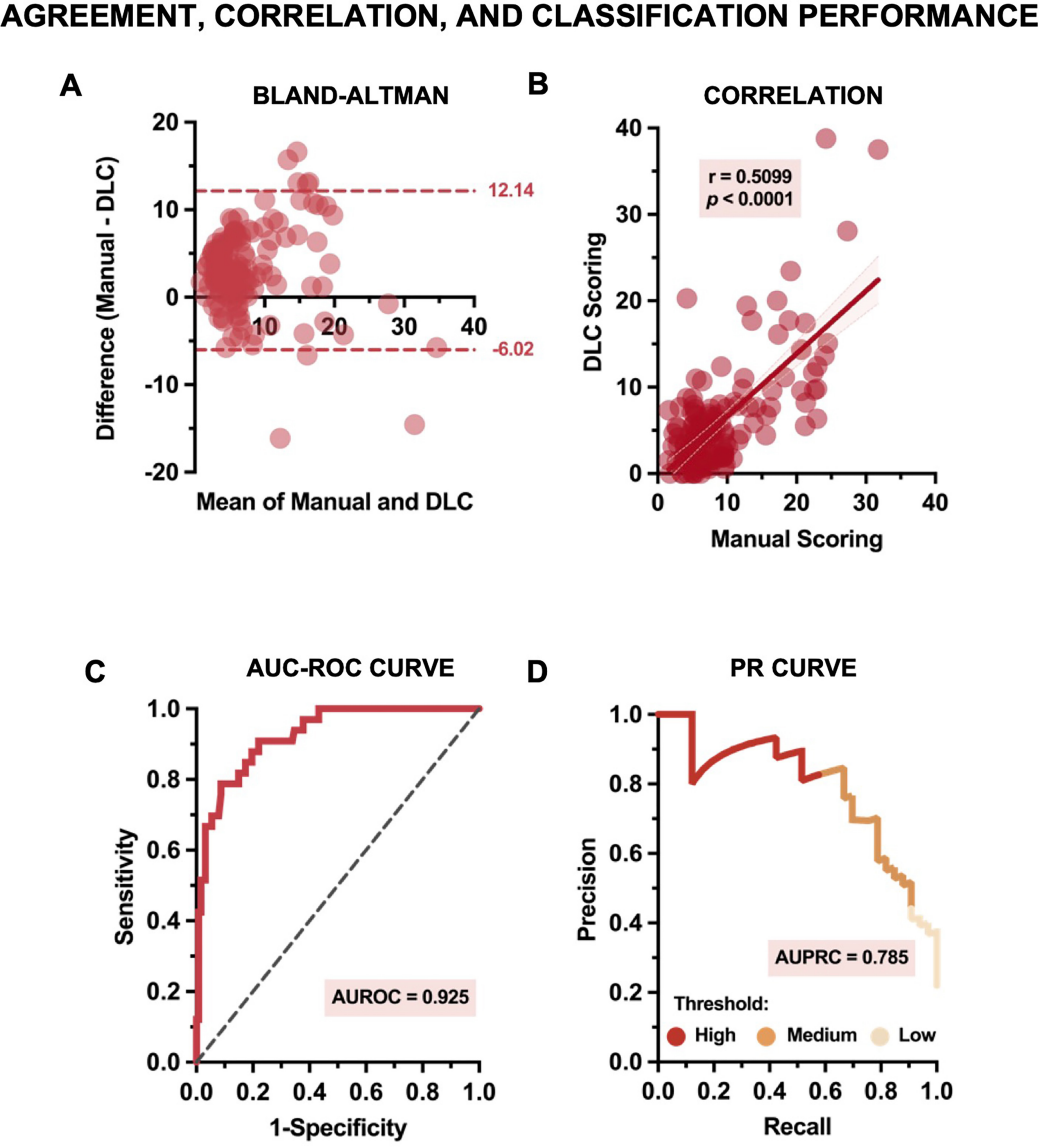
DeepLabCut (DLC) provides reliable automated misstep detection. **(A)** Bland-Altman plot comparing DLC and manual scoring of missteps, shows the mean bias was 3 percentage points, indicating that DLC slightly underestimates missed steps relative to manual scoring. **(B)** Spearman correlation between manual and DLC scoring showing a moderate positive association. **(C)** Receiver operating characteristic (ROC) curve for misstep detection using a manual threshold of 11.56%, yielding AUROC = 0.925, and **(D)** Precision-recall (PR) curve for the same threshold yielded AUPRC = 0.785. *P*-values < 0.05 were considered statistically significant. All analyses were performed using the percentage of missteps for both the affected and unaffected forepaws pooled across all testing days, *n* = 160.

To further validate the model, we evaluated agreement, correlation, and misstep detection performance between manual and DLC-based scoring as secondary endpoints. Agreement between DLC and manual scoring was evaluated using Bland-Altman analysis and ICC. The Bland-Altman analysis revealed a mean bias of 3 percentage points of missed steps, indicating that the DLC method underestimates missed steps by an average of 3 percentage points compared to manual scoring (Figure 5A). The standard deviation of the differences was 4.63 percentage points, with 95% limits of agreement ranging from −6.02 pp to 12.14 pp. The histogram of differences between manual and DLC-based scoring showed a mean discrepancy of approximately 3 percentage points between the two scoring approaches (95% CI: 2.34–3.78), with manual scoring generally yielding higher misstep percentages than the DLC-based method (Supplementary Figure 4). The ICC, calculated using a two-way mixed, single-rater, absolute agreement model, was 0.62 (*F* (159, 14.9) = 5.73, *p* < 0.0002; 95% CI: 0.32–0.78), reflecting moderate agreement.

Spearman correlation analysis showed a moderate positive association between manual scoring and DLC scoring (*r* = 0.51, 95% CI: 0.38–0.62, Figure 5B). The correlation was statistically significant (*p* < 0.0001), confirming that the observed relationship is unlikely to be due to chance.

For misstep detection, manual scoring was binarized using a threshold of 11.56% missteps. At this threshold, the DLC model achieved an AUROC of 0.925 and an AUPRC of 0.785, indicating good overall discriminative performance (Figures 5C, D). Optimal detection, determined via the Youden index (0.701), corresponded to a threshold of 7.63% missteps, with a specificity of 0.913 and sensitivity of 0.788. These results demonstrate that DLC provides automated misstep detection, with a good balance between avoiding false positives and correctly identifying real missteps.

## 4 Discussion

The primary aim of this study was to assess the performance of the commercially available Automatic Ladder Test (BIO-LADDERTEST, BIOSEB) for detecting post-stroke motor impairments in mice and to determine its suitability as a behavioral assessment tool. In parallel, we developed a DLC-based automated scoring model for the grid walking test following photothrombotic stroke. Whereas the automated ladder test exhibited limited sensitivity to stroke-induced impairments, the DLC-based approach documented motor deficit, establishing deep learning-based behavioral analysis as a scalable and sensitive strategy for automated post-stroke phenotyping.

The horizontal ladder test was originally developed about two decades ago by Metz and Whishaw to evaluate motor impairments following cortical and subcortical lesions (Metz and Whishaw, 2002). Since its introduction, this test has proven to be a sensitive tool for assessing motor dysfunction across various experimental models, including spinal cord injury (Zhan et al., 2023), osteoarthritis (Chavez et al., 2019), and age-related motor performance deficits in rodents (Metz and Whishaw, 2002; Soleman et al., 2010). Over time, the horizontal ladder test has also become widely used for evaluating motor dysfunction following a stroke. It was used in detecting post-stroke motor impairments, both in early (Soleman et al., 2010; Livingston-Thomas et al., 2013; Cappelli et al., 2021; Li et al., 2022; Inoue et al., 2023) and chronic phases of stroke (Metz and Whishaw, 2002; Rust et al., 2019; Weber et al., 2022) in various models, including photothrombosis.

A key feature of human post-stroke pathology is hemiparesis–a condition characterized by unilateral motor deficit due to infarction in the contralateral hemisphere (Noskin et al., 2008), which is mimicked in most rodent stroke models. Given this pathophysiological relevance, an ideal test for assessing post-stroke motor dysfunction/recovery should specifically target the limb contralateral to the injury, rather than evaluating the overall motor performance to ensure that functional impairments are attributed to stroke-related deficit rather than compensatory strategies or generalized motor impairments (Krakauer, 2006; Jones and Adkins, 2015). Because of this, the spontaneous forelimb behavioral tasks, like the ladder and grid walking tests, are ideal to evaluate motor function in experimental studies.

The BIOSEB automated ladder test is a proprietary instrument equipped with two rows of infrared beams–one positioned above and the other below the ladder rungs–to detect paw placement errors, such as slips or missteps between bars. Additionally, a high-speed camera captures the paw positions of the animal from underneath, allowing for precise tracking of all four limbs, speed, and shape of the animal. This setup enables differentiation between forelimb and hindlimb movements, as well as right-left asymmetries in motor function. Given these characteristics, the automated ladder test appears to be a perfect tool for unbiased and time-effective analysis of unilateral motor deficits in rodent models of neurological disorders. However, contrary to our expectation, the results of this study indicate that the automated instrument did not detect significant alterations in front paw missteps in mice after stroke. Notably, the same animals tested in parallel in the grid walking test showed substantial motor impairment, which persisted for at least 14 days after stroke.

To better understand the results of the ladder test in our study, we examined key experimental parameters that might influence the outcomes. These parameters included the regularity of the rung pattern, the number of trials, and the specific metrics assessed (e.g., misstep or foot fault scale) (Metz and Whishaw, 2002; Antonow-Schlorke et al., 2013). Analysis of rung patterns randomly changed between experimental days in our study did not lead to significant differences in misstep counts between sham and stroke-operated mice (our presented data includes all variations of rung patterns). Notably, both regular (Hunsaker et al., 2011; Hines and Haydon, 2013) and irregular (Livingston-Thomas et al., 2013; Cappelli et al., 2021; Li et al., 2022; Inoue et al., 2023) patterns of rungs have been used in previous studies, though irregular spacing appears to be recommended for running the test (Metz and Whishaw, 2002; Soleman et al., 2010). Next, we assessed whether the number of crossings per trial may affect the test outcomes. Comparison of front paw missteps between 5 (first and last) and 15 crossings in the ladder showed no significant difference, suggesting that the absence of detectable motor deficit was not due to the number of crossings per trial performed. Notably, prior rodent stroke studies using the conventional ladder test have applied a wide range of crossings per trial, e.g., two (Soleman et al., 2010), three (Weber et al., 2022), six (Rust et al., 2019), ten (Inoue et al., 2023), and documented a significant motor deficit after stroke.

When assessing motor deficits using the horizontal ladder test, Metz and Whishaw (Metz and Whishaw, 2002) suggested two interchangeably used endpoints: (a) misstep ratio, which quantifies the proportion of missteps relative to total steps, and (b) foot fault score, a seven-category scale that provides a detailed assessment of paw placement accuracy. The foot fault scale classifies errors into seven categories: total miss, deep slip, slight slip, replacement, correction, partial placement, and correct placement. A total miss occurs when a limb falls between rungs, resulting in a loss of balance; a deep slip involves a limb slipping off a rung while bearing weight, leading to a fall; and a slight slip refers to a limb sliding off a rung without causing a fall, allowing the subject to continue walking (Metz and Whishaw, 2002). According to the automated ladder test instrument’s user manual, the system defines an error as occurring when “the paw slips between bars and triggers the lower infrared beams.” This classification likely corresponds to the three most severe categories of missteps defined by Metz and Whishaw (Metz and Whishaw, 2002), which we reported as the misstep ratio. The latter readout is well-established for quantifying motor impairments, demonstrating a strong correlation with the foot fault score (Antonow-Schlorke et al., 2013), and has been widely utilized in previous studies (Hines and Haydon, 2013; Livingston-Thomas et al., 2013; Cappelli et al., 2021; Inoue et al., 2023), so it is surprising that the automated scoring lacked sensitivity in detecting motor deficit. One possible reason for the discrepancy observed in the BIOSEB ladder test results may be its limited sensitivity to subtle or very quick missteps. Minor or very quick paw placement errors might not interrupt the infrared beam and therefore remain undetected by the system. Implementing a multi-level, e.g., seven-category scale to assess paw placement accuracy could further enhance detection sensitivity and provide a more nuanced evaluation of motor performance. Several additional factors may also have contributed to the negative findings. These include the threshold settings of the infrared sensors, although we attempted to minimize this issue by positioning the ladder at the lowest available detection level. Furthermore, the relatively low frequency of missteps may reflect a ceiling effect (the task was insufficiently challenging to discriminate between groups), or the adoption of compensatory locomotor strategies (Metz and Whishaw, 2002). However, to maximize task difficulty and sensitivity, ladder testing was performed using an irregular rung arrangement. We also examined performance under regular rung spacing and analyzed early and late trials separately; however, these additional analyses also did not reveal significant group differences. Given the observed variability and sample size of the ladder test dataset, the present findings argue against the presence of a significant motor deficit (please see *Cohen’s f* effect size estimates in Supplementary Table 2). Nevertheless, more subtle motor impairment may remain undetected due to the sensitivity limits of the current automated instrument. Finally, we examined whether the instrument provides reliable results for the steps per limb. We found comparable step counts between sham and stroke groups, as well as between affected and unaffected forelimbs, with two steps taken per second. This suggests that the automated step detection was consistent and reliable, thus confirming the inaccurate misstep scoring as the likely reason for the instrument’s failure to detect unilateral motor impairments.

While previous studies have successfully used the BIOSEB automated ladder test instrument to assess motor function across various disease models, including Tcf4 haploinsufficient mice (Kennedy et al., 2016), osteoarthritis (Chavez et al., 2019), middle-aged mice (Zhu et al., 2021), and the transient MCAO stroke model (Wang et al., 2024), these studies analyzed missteps aggregated across all four limbs rather than unilateral deficits. This distinction may explain why, in our study focused on forepaw-specific impairments, the BIOSEB system did not detect significant group differences. Importantly, our findings apply specifically to the current configuration of the BIOSEB instrument, its default error-detection approach, and the experimental protocol used in the present study.

Recent advances in AI have facilitated the development of deep learning-based programs for highly sensitive and specific behavioral quantification. One such tool is DLC (Mathis et al., 2018), a markerless pose-estimation software utilizing deep neural networks. The DLC-assisted model has been successfully applied to quantify missteps in the horizontal ladder test (Aljovic et al., 2022; Piotrowski et al., 2024), including studies investigating motor dysfunction and recovery after photothrombotic stroke (Rust et al., 2019; Weber et al., 2022). In a study by Weber et al. (2022), DLC enabled the detection of significant unilateral increases in missteps by post-stroke mice in the ladder test over the course of 3 weeks. Additionally, the software identified biologically relevant alterations in locomotor profiles following stroke, including synchronization between the paws and step cycle while crossing the ladder. The model created by Weber and co-authors (Weber et al., 2022) has emerged as a compelling alternative to the commercial automated ladder test instrument used in our study. Not only does it enable robust and reliable detection of motor dysfunction, but it also offers a substantially more cost-effective solution. Building on this major advancement, we wanted to evaluate whether a DLC-assisted model could similarly perform locomotor analysis and quantify missteps in the grid walking test. To enable precise classification of limb-specific deficits, our model was trained to distinguish both the anterior-posterior orientation and the lateral (left-right) positioning of the mouse. To achieve this, we annotated eight anatomical landmarks and trained the network on over 4,000 frames capturing limb missteps from diverse perspectives. Evaluation of our model showed that the loss converged near zero after approximately 500,000 iterations and achieved labeling accuracy with minimal millimeter-level error between manual and predicted annotations, consistent with the performance of previously developed DLC-based models (Forys et al., 2020; Laurence-Chasen et al., 2020; Weber et al., 2022).

Our DLC-based training approach yielded a reliable model with high labeling accuracy, capable of detecting affected forepaw missteps over the course of the study in a pattern consistent with blinded manual scoring. Comparison of stroke and sham groups, both manual and DLC-based analyses, revealed a ∼3-fold increase in missteps in stroke-affected animals. Similarly, compared to their baseline motor performance, stroke animals showed approximately a threefold increase in missteps on PSD3 and a twofold increase on PSD14 across both scoring methods. The divergence occurred on PSD7, where the blinded manual scoring revealed a 2.7-fold increase relative to baseline, while the DLC-based method showed a 3-fold increase. Importantly, the best practices recommend expressing motor deficits in the grid walking test as the ratio of missteps to steps, which accounts for the natural decline in exploratory behavior throughout longitudinal studies and varies in baseline activity levels between individual animals (Pisula and Siegel, 2005; Syeara et al., 2023). Our additional analyses further confirmed that the DLC-based approach provides robust and reliable misstep detection. Bland-Altman analysis revealed a minimal mean bias of 3 percentage points, indicating that DLC slightly underestimates missteps compared with manual scoring. The ICC indicated moderate agreement between the two methods, while Spearman correlation demonstrated a statistically significant moderate positive relationship between manual scoring and DLC scoring. In addition, the DLC model showed strong discriminative performance, accurately identifying true missteps while maintaining a low rate of false positives. In our analysis, manual step counts closely matched those generated by the DLC-based model, demonstrating near-identical values, confirming the validity of DLC-derived coordinates and supporting its utility for detecting general locomotor activity in mice, consistent with prior studies (Sheppard et al., 2022).

It is noteworthy that there have been other attempts to automate foot-fault quantification in rodents in the past. Commercially available instruments include FootFaultScan [also known as Parallel Rod Floor Test; Clever System (Kamens and Crabbe, 2007)], LadderScan [Clever System (Wang et al., 2018)], MotoRater [TSE Systems (Zörner et al., 2010)], and ErasmusLadder [New Biotechnology (Almeida et al., 2020)], all of which resemble the ladder rung test to some degree. Importantly, LadderScan, MotoRater, and ErasmusLadder can differentiate between left and right limbs and, by that, quantify the affected and unaffected limb function. On the contrary, FootFaultScan, which has some resemblance to the grid walking test too (rectangular platform with a parallel rod floor, not a grid), quantifies total foot faults from all limbs without distinguishing between them. To our knowledge, currently, there are no reports about the automation of the traditional grid walking test that has been adopted and widely used in the last two decades (Syeara et al., 2023). In one publication, a computerized device combining the features of the ladder test (long corridor) and the grid walking test (a grid floor) was reported (Prakriya et al., 1993); however, similar to the FootFaultScan, it quantifies total foot faults without distinguishing between affected and unaffected sides. Although deep-learning approaches have been applied to treadmill- (Aljovic et al., 2022) and walkway-based (Molina et al., 2023) gait assessment systems, these paradigms primarily quantify global gait parameters such as stride length and swing duration. In contrast, the grid walking test specifically challenges precise paw placement and sensorimotor integration, making it particularly sensitive to subtle forelimb deficits following focal cortical stroke. This is especially relevant for post-stroke recovery studies, as the Stroke Recovery and Rehabilitation Roundtable guidelines (Corbett et al., 2017) specifically recommend the grid walking test for the longitudinal assessment of forelimb motor function in rodent stroke models.

The success of our DLC-based model in detecting post-ischemic motor dysfunction underscores the growing potential of AI-powered tools as highly accurate and time-efficient platforms for behavioral phenotyping (von Ziegler et al., 2021). The rapid explosion of studies employing AI for behavioral analysis in diverse animal models, including *Drosophila* (Günel et al., 2019; Hu et al., 2023), zebrafish (Braun et al., 2024; Gendelev et al., 2024), rodents (Bordes et al., 2023; Wang et al., 2023; Aldarondo et al., 2024), and non-human primates (Ha et al., 2024), reflects a paradigm shift within the scientific community toward AI-driven methodologies in neuroscience (von Ziegler et al., 2021). These tools are also being added to the product lines of companies that provide automated behavioral testing systems (Conduct Science, 2025) and are now used in early-stage research by major biopharmaceutical companies (Digital in Vivo Alliance [DIVA], 2025). AI-based platforms offer several key advantages: they eliminate human bias, enhance reproducibility, enable the analysis of complex behavioral endpoints, and support both cost-effective and high-throughput experimental designs. Even though commercially available systems, such as the automated ladder test from BIOSEB, offer other benefits, including real-time data acquisition and easy-to-use software without the need for programming expertise or model training, DLC-driven approaches provide unparalleled flexibility and substantially lower cost. Although the present study did not directly quantify workflow duration, labor requirements, or cost-effectiveness, DLC-based analysis has the potential to reduce active investigator involvement by shifting portions of behavioral assessment from manual scoring to passive computational processing. While video acquisition, model training, computational processing, and quality control require dedicated infrastructure and oversight, conventional manual scoring often requires continuous investigator involvement and frame-by-frame evaluation. Once trained, a DLC pipeline can enable automated processing of large datasets, potentially improving scalability and reducing investigator workload. Future studies should perform detailed workflow comparisons, similar to those reported previously (Weber et al., 2022), to quantitatively assess the time and resource requirements of DLC-based versus conventional manual approaches. Furthermore, automated analysis applies identical criteria across all recordings, reducing potential observer-dependent variability, fatigue-related errors, and unconscious bias, while enhancing reproducibility and standardization across experiments.

Beyond preclinical animal studies, AI-driven methodologies are becoming integrated into clinical practice, including the diagnosis and management of ischemic stroke. Advanced neuroimaging enhanced by AI algorithms has significantly improved diagnostic precision, enabling earlier detection of ischemic events, identification of relevant biological biomarkers, prediction of clinical outcomes, and optimization of individualized rehabilitation protocols (Ding et al., 2020; Dresser and Kohn, 2023; Kopalli et al., 2025). DLC has also been successfully translated to clinical studies for high-resolution, non-invasive analysis of gait patterns (Panconi et al., 2025), sit-to-stand transitions (Milone et al., 2024), and complex motor behaviors such as speech (Wrench and Balch-Tomes, 2022). A recent meta-analysis demonstrated that AI-assisted rehabilitation significantly enhances conventional strategies in improving post-stroke functional outcomes (Antoni et al., 2025) and has the potential to be integrated into remote patient monitoring and become standard-of-care in neurorehabilitation (Kopalli et al., 2025).

Notably, our study has several limitations. First, experiments were conducted exclusively in male CD-1 mice with narrow age range (8–10 weeks), which limits generalizability across sex, strain, and age. Second, although the DLC model was evaluated using repeated frame-level train/test splits with consistent performance across shuffles, all data originated from a single experimental cohort, and no independent external validation cohort at the animal or study level was included. Nevertheless, the training dataset was constructed using only a subset of animals (10 of the 20 animals included in the study), and annotated frames were randomly selected across four different time points rather than being derived from complete recordings of individual animals. Another limitation of our study is that the trained model does not quantify the severity of missteps or include kinematic parameters. Such an “advanced grid walking test” which incorporates parameters like step and misstep length, depth, duration, velocity, trajectory, paw placement angle, inter-limb coordination, and joint kinematics, could potentially enhance the accuracy of motor performance and recovery assessment. Future versions of the DLC-based grid walking test could include these additional parameters with a stronger focus on the severity of each misstep. This could minimize threshold asymmetry and improve the system’s sensitivity to identify both mild and severe motor deficits and recovery. Finally, the manual scoring in our study was performed by a single blinded investigator; therefore, inter-rater reliability could not be assessed, and the potential influence of scorer-dependent variability cannot be excluded.

In summary, our observations show that the commercially available automated ladder test (BIO-LADDERTEST, BIOSEB, France) lacked the sensitivity required to consistently detect unilateral sensorimotor deficits in a mouse model of photothrombotic stroke. By contrast, we have developed for the first time an automated DLC-based model to assess missteps in the grid walking test, and our deep learning-based model reliably identified motor impairment in the same stroke animals. These results highlight the reproducibility and objectivity of AI-powered behavioral analysis, and future studies may explore whether integrating machine learning-based detection into the automated ladder test could enhance its performance. Collectively, our data support the growing potential of deep learning-driven platforms to enhance methodological standards in experimental stroke research.

## Data Availability

The original contributions presented in this study are included in this article/Supplementary material, further inquiries can be directed to the corresponding author.

## Supplementary material

Supplementary Video 1 Representative training video with front-view annotations.

Supplementary Video 2 Representative training video with back-view annotations.

## References

[B1] Al Shoyaib A. Alamri F. F. Biggers A. Karamyan S. T. Arumugam T. V. Ahsan F. et al. (2021a). Delayed exercise-induced upregulation of angiogenic proteins and recovery of motor function after photothrombotic stroke in mice. *Neuroscience* 461 57–71. 10.1016/J.NEUROSCIENCE.2021.02.023 33667592 PMC8044041

[B2] Al Shoyaib A. Alamri F. F. Syeara N. Jayaraman S. Karamyan S. T. Arumugam T. V. et al. (2021b). The effect of histone deacetylase inhibitors panobinostat or entinostat on motor recovery in mice after ischemic stroke. *Neuromol. Med.* 23 471–484. 10.1007/S12017-021-08647-1 33590407 PMC8364572

[B3] Alamri F. F. Karamyan S. T. Karamyan V. T. (2023). A low-budget photothrombotic rodent stroke model. *Methods Mol. Biol.* 2616 21–28. 10.1007/978-1-0716-2926-0_3 36715924

[B4] Alamri F. F. Shoyaib A. Al, Biggers A. Jayaraman S. Guindon J. et al. (2018). Applicability of the grip strength and automated von Frey tactile sensitivity tests in the mouse photothrombotic model of stroke. *Behav. Brain Res.* 336 250–255. 10.1016/J.BBR.2017.09.008 28893552

[B5] Alamri F. Al Shoyaib A. Syeara N. Paul A. Jayaraman S. Karamyan S. et al. (2021). Delayed atomoxetine or fluoxetine treatment coupled with limited voluntary running promotes motor recovery in mice after ischemic stroke. *Neural Regen. Res.* 16 1244–1251. 10.4103/1673-5374.301031 33318401 PMC8284259

[B6] Aldarondo D. Merel J. Marshall J. D. Hasenclever L. Klibaite U. Gellis A. et al. (2024). A virtual rodent predicts the structure of neural activity across behaviours. *Nature* 632 594–602. 10.1038/S41586-024-07633-4 38862024 PMC12080270

[B7] Aljovic A. Zhao S. Chahin M. de la Rosa C. Van Steenbergen V. Kerschensteiner M. et al. (2022). A deep learning-based toolbox for Automated limb motion analysis (ALMA) in murine models of neurological disorders. *Commun. Biol.* 5:131. 10.1038/S42003-022-03077-6 35169263 PMC8847458

[B8] Almeida L. E. F. Wang L. Kamimura S. Zerfas P. M. Smith M. L. Neto O. L. A. et al. (2020). Locomotor mal-performance and gait adaptability deficits in sickle cell mice are associated with vascular and white matter abnormalities and oxidative stress in cerebellum. *Brain Res.* 1746:146968. 10.1016/J.BRAINRES.2020.146968 32533970 PMC7483603

[B9] Antoni T. Benedictus B. Putra S. E. (2025). Functional and motoric outcome of AI-assisted stroke rehabilitation: A meta-analysis of randomized controlled trials. *Brain Disord.* 18:100224. 10.1016/J.DSCB.2025.100224

[B10] Antonow-Schlorke I. Ehrhardt J. Knieling M. (2013). Modification of the ladder rung walking task-new options for analysis of skilled movements. *Stroke Res. Treat.* 2013, 1–11. 10.1155/2013/418627 23577278 PMC3610362

[B11] Automatic Ladder Test [BIOSEB],. (2025). Available online at: https://bioseb.com/en/activity-motor-control-coordination/1853-automatic-ladder-test.html (accessed August 18, 2025).

[B12] Balkaya M. Kröber J. M. Rex A. Endres M. (2013). Assessing post-stroke behavior in mouse models of focal ischemia. *J. Cereb. Blood Flow Metab.* 33 330–338. 10.1038/JCBFM.2012.185 23232947 PMC3587814

[B13] Barios J. A. Pisarchyk L. Fernandez-Garcia L. Barrio L. C. Ramos M. Martinez-Murillo R. et al. (2016). Long-term dynamics of somatosensory activity in a stroke model of distal middle cerebral artery oclussion. *J. Cereb. Blood Flow Metab.* 36 606–620. 10.1177/0271678X15606139 26661150 PMC4794092

[B14] Bordes J. Miranda L. Reinhardt M. Narayan S. Hartmann J. Newman E. L. et al. (2023). Automatically annotated motion tracking identifies a distinct social behavioral profile following chronic social defeat stress. *Nat. Commun.* 14:4319. 10.1038/S41467-023-40040-3 37463994 PMC10354203

[B15] Braun D. Rosenberg A. M. Rabaniam E. Haruvi R. Malamud D. Barbara R. et al. (2024). High-resolution tracking of unconfined zebrafish behavior reveals stimulatory and anxiolytic effects of psilocybin. *Mol. Psychiatry* 29 1046–1062. 10.1038/S41380-023-02391-7 38233467 PMC11176078

[B16] Britsch D. R. S. Syeara N. Stowe A. M. Karamyan V. T. (2023). Rodent stroke models to study functional recovery and neural repair. *Methods Mol. Biol.* 2616 3–12. 10.1007/978-1-0716-2926-0_1 36715922

[B17] Cappelli J. Khacho P. Wang B. Sokolovski A. Bakkar W. Raymond S. et al. (2021). Glycine-induced NMDA receptor internalization provides neuroprotection and preserves vasculature following ischemic stroke. *iScience* 25:103539. 10.1016/J.ISCI.2021.103539 34977503 PMC8689229

[B18] Casals J. B. Pieri N. C. G. Feitosa M. L. T. Ercolin A. C. M. Roballo K. C. S. Barreto R. S. N. et al. (2011). The use of animal models for stroke research: A review. *Comp. Med.* 61 305–313. 22330245 PMC3155396

[B19] Chavez R. D. Sohn P. Serra R. (2019). Prg4 prevents osteoarthritis induced by dominant-negative interference of TGF-ß signaling in mice. *PLoS One* 14:e0210601. 10.1371/journal.pone.0210601 30629676 PMC6328116

[B20] Clarkson A. N. López-Valdés H. E. Overman J. J. Charles A. C. Brennan K. C. Thomas Carmichael S. (2013). Multimodal examination of structural and functional remapping in the mouse photothrombotic stroke model. *J. Cereb. Blood Flow Metab.* 33 716–723. 10.1038/JCBFM.2013.7 23385201 PMC3652691

[B21] Conduct Science,. (2025). Available online at: https://conductscience.com (accessed August 18, 2025).

[B22] Corbett D. Carmichael S. T. Murphy T. H. Jones T. A. Schwab M. E. Jolkkonen J. et al. (2017). Enhancing the alignment of the preclinical and clinical stroke recovery research pipeline: Consensus-based core recommendations from the Stroke Recovery and Rehabilitation Roundtable translational working group. *Int. J. Stroke* 12 462–471. 10.1177/1747493017711814 28697710

[B23] Digital in Vivo Alliance [DIVA],. (2025). Available online at: https://diva.bio (accessed August 18, 2025).

[B24] Ding L. Liu C. Li Z. Wang Y. (2020). Incorporating artificial intelligence into stroke care and research. *Stroke* 51 E351–E354. 10.1161/STROKEAHA.120.031295 33106108

[B25] Doeppner T. R. Kaltwasser B. Bähr M. Hermann D. M. (2014). Effects of neural progenitor cells on post-stroke neurological impairment-a detailed and comprehensive analysis of behavioral tests. *Front. Cell. Neurosci.* 8:338. 10.3389/FNCEL.2014.00338 25374509 PMC4205824

[B26] Dresser L. P. Kohn M. A. (2023). Artificial intelligence and the evaluation and treatment of stroke. *Dela. J. Public Health* 9 82–84. 10.32481/DJPH.2023.08.014 37701474 PMC10494798

[B27] Feigin V. L. Stark B. A. Johnson C. O. Roth G. A. Bisignano C. Abady G. G. et al. (2021). Global, regional, and national burden of stroke and its risk factors, 1990-2019: A systematic analysis for the Global Burden of Disease Study 2019. *Lancet Neurol.* 20 1–26. 10.1016/S1474-4422(21)00252-0 34487721 PMC8443449

[B28] Forys B. J. Xiao D. Gupta P. Murphy T. H. (2020). Real-time selective markerless tracking of forepaws of head fixed mice using deep neural networks. *eNeuro* 7 1–14. 10.1523/ENEURO.0096-20.2020 32409507 PMC7307631

[B29] Friard O. Gamba M. (2016). BORIS: A free, versatile open-source event-logging software for video/audio coding and live observations. *Methods Ecol. Evol.* 7 1325–1330. 10.1111/2041-210X.12584

[B30] Gendelev L. Taylor J. Myers-Turnbull D. Chen S. McCarroll M. N. Arkin M. R. et al. (2024). Deep phenotypic profiling of neuroactive drugs in larval zebrafish. *Nat. Commun.* 15:9955. 10.1038/S41467-024-54375-Y 39551797 PMC11570628

[B31] Günel S. Rhodin H. Morales D. Campagnolo J. Ramdya P. Fua P. (2019). DeepFly3D, a deep learning-based approach for 3D limb and appendage tracking in tethered, adult Drosophila. *Elife* 8:e48571. 10.7554/ELIFE.48571 31584428 PMC6828327

[B32] Ha L. J. Yeo H. G. Kim Y. G. Baek I. Baeg E. Lee Y. H. et al. (2024). Hypothalamic neuronal activation in non-human primates drives naturalistic goal-directed eating behavior. *Neuron* 112 2218–2230.e6. . 10.1016/J.NEURON.2024.03.029 38663401

[B33] Hines D. J. Haydon P. G. (2013). Inhibition of a SNARE-sensitive pathway in astrocytes attenuates damage following stroke. *J. Neurosci.* 33 4234–4240. 10.1523/JNEUROSCI.5495-12.2013 23467341 PMC3699341

[B34] Hu Y. Ferrario C. R. Maitland A. D. Ionides R. B. Ghimire A. Watson B. et al. (2023). LabGym: Quantification of user-defined animal behaviors using learning-based holistic assessment. *Cell Rep. Methods* 3:100415. 10.1016/J.CRMETH.2023.100415 37056376 PMC10088092

[B35] Hunsaker M. R. von Leden R. E. Ta B. T. Goodrich-Hunsaker N. J. Arque G. Kim K. et al. (2011). Motor deficits on a ladder rung task in male and female adolescent and adult CGG knock-in mice. *Behav. Brain Res.* 222 117–121. 10.1016/J.BBR.2011.03.039 21440572 PMC3095688

[B36] Inoue K. Asaka M. Lee S. Ishikawa K. Yanagihara D. (2023). Gait disorders induced by photothrombotic cerebellar stroke in mice. *Sci. Rep.* 13:15805. 10.1038/S41598-023-42817-4 37737224 PMC10516889

[B37] Jayaraman S. Al Shoyaib A. Kocot J. Villalba H. Alamri F. F. Rashid M. et al. (2020). Peptidase neurolysin functions to preserve the brain after ischemic stroke in male mice. *J. Neurochem.* 153 120–137. 10.1111/JNC.14864 31486527 PMC7056597

[B38] Jones T. A. Adkins D. L. (2015). Motor system reorganization after stroke: Stimulating and training toward perfection. *Physiology* 30 358–370. 10.1152/PHYSIOL.00014.2015 26328881 PMC4556825

[B39] Joy M. T. Ben Assayag E. Shabashov-Stone D. Liraz-Zaltsman S. Mazzitelli J. Arenas M. et al. (2019). CCR5 is a therapeutic target for recovery after stroke and traumatic brain injury. *Cell* 176 1143–1157.e13. 10.1016/J.CELL.2019.01.044 30794775 PMC7259116

[B40] Kamens H. M. Crabbe J. C. (2007). The parallel rod floor test: A measure of ataxia in mice. *Nat. Protoc.* 2 277–281. 10.1038/NPROT.2007.19 17406586

[B41] Karamyan V. T. (2023). Clinically applicable experimental design and considerations for stroke recovery preclinical studies. *Methods Mol. Biol.* 2616 369–377. 10.1007/978-1-0716-2926-0_25 36715946

[B42] Kennedy A. J. Rahn E. J. Paulukaitis B. S. Savell K. E. Kordasiewicz H. B. Wang J. et al. (2016). Tcf4 regulates synaptic plasticity, DNA methylation, and memory function. *Cell Rep.* 16 2666–2685. 10.1016/J.CELREP.2016.08.004 27568567 PMC5710002

[B43] Kishi T. Kobayashi K. Sasagawa K. Sakimura K. Minato T. Kida M. et al. (2025). Automated analysis of a novel object recognition test in mice using image processing and machine learning. *Behav. Brain Res.* 476:115278. 10.1016/J.BBR.2024.115278 39357746

[B44] Kopalli S. R. Shukla M. Jayaprakash B. Kundlas M. Srivastava A. Jagtap J. et al. (2025). Artificial intelligence in stroke rehabilitation: From acute care to long-term recovery. *Neuroscience* 572 214–231. 10.1016/J.neuroscience.2025.03.017 40068721

[B45] Krakauer J. W. (2006). Motor learning: Its relevance to stroke recovery and neurorehabilitation. *Curr. Opin. Neurol.* 19 84–90. 10.1097/01.WCO.0000200544.29915.CC 16415682

[B46] Laurence-Chasen J. D. Manafzadeh A. R. Hatsopoulos N. G. Ross C. F. Arce-Mcshane F. I. (2020). Integrating XMALab and DeepLabCut for high-throughput XROMM. *J. Exp. Biol.* 223:226720. 10.1242/JEB.226720 32665442 PMC7490514

[B47] Li S. S. Xing X. X. Hua X. Y. Zhang Y. W. Wu J. J. Shan C. L. et al. (2022). Alteration of brain functional networks induced by electroacupuncture stimulation in rats with ischemia-reperfusion: An independent component analysis. *Front. Neurosci.* 16:958804. 10.3389/FNINS.2022.958804 35992929 PMC9382119

[B48] Li Y. Zhang J. (2021). Animal models of stroke. *Animal Model. Exp. Med.* 4 204–219. 10.1002/AME2.12179 34557647 PMC8446711

[B49] Livingston-Thomas J. M. Hume A. W. Doucette T. A. Tasker R. A. (2013). A novel approach to induction and rehabilitation of deficits in forelimb function in a rat model of ischemic stroke. *Acta Pharmacol. Sin.* 34 104–112. 10.1038/APS.2012.106 23103624 PMC4086495

[B50] Mathis A. Mamidanna P. Cury K. M. Abe T. Murthy V. N. Mathis M. W. et al. (2018). DeepLabCut: Markerless pose estimation of user-defined body parts with deep learning. *Nat. Neurosci.* 21 1281–1289. 10.1038/S41593-018-0209-Y 30127430

[B51] Matur A. V. Candelario-Jalil E. Paul S. Karamyan V. T. Lee J. D. Pennypacker K. et al. (2023). Translating animal models of ischemic stroke to the human condition. *Transl. Stroke Res.* 14 842–853. 10.1007/S12975-022-01082-9 36125734

[B52] Metz G. A. Whishaw I. Q. (2002). Cortical and subcortical lesions impair skilled walking in the ladder rung walking test: A new task to evaluate fore- and hindlimb stepping, placing, and co-ordination. *J. Neurosci. Methods* 115 169–179. 10.1016/S0165-0270(02)00012-2 11992668

[B53] Metz G. A. Whishaw I. Q. (2009). The ladder rung walking task: A scoring system and its practical application. *J. Vis. Exp.* 28:1204. 10.3791/1204 19525918 PMC2796662

[B54] Milone D. Longo F. Merlino G. De Marchis C. Risitano G. D’Agati L. (2024). MocapMe: DeepLabCut-enhanced neural network for enhanced markerless stability in sit-to-stand motion capture. *Sensors* 24:3022. 10.3390/S24103022 38793876 PMC11125421

[B55] Molina L. A. Milla-Cruz J. J. Ghavasieh Z. Kim L. H. Cheng N. Whelan P. J. (2023). High-throughput gait acquisition system for freely moving mice. *J. Neurophysiol.* 130 1081–1091. 10.1152/jn.00133.2023 37728487

[B56] Noskin O. Krakauer J. W. Lazar R. M. Festa J. R. Handy C. O’Brien K. A. et al. (2008). Ipsilateral motor dysfunction from unilateral stroke: Implications for the functional neuroanatomy of hemiparesis. *J. Neurol. Neurosurg. Psychiatry* 79 401–406. 10.1136/JNNP.2007.118463 17635970

[B57] Nowak B. Rogujski P. Guzman R. Walczak P. Andrzejewska A. Janowski M. (2023). Animal models of focal ischemic stroke: Brain size matters. *Front. Stroke* 2:1165231. 10.3389/FSTRO.2023.1165231 41541099 PMC12802671

[B58] Panconi G. Grasso S. Guarducci S. Mucchi L. Minciacchi D. Bravi R. (2025). DeepLabCut custom-trained model and the refinement function for gait analysis. *Sci. Rep.* 15:2364. 10.1038/S41598-025-85591-1 39824885 PMC11742404

[B59] Piotrowski D. Clemensson E. K. H. Nguyen H. P. Mark M. D. (2024). Phenotypic analysis of ataxia in spinocerebellar ataxia type 6 mice using DeepLabCut. *Sci. Rep.* 14:8571. 10.1038/S41598-024-59187-0 38609436 PMC11014858

[B60] Pisula W. Siegel J. (2005). Exploratory behavior as a function of environmental novelty and complexity in male and female rats. *Psychol. Rep.* 97 631–638. 10.2466/PR0.97.2.631-638 16342593

[B61] Prakriya M. McCabe P. M. Holets V. R. (1993). A computerized grid walking system for evaluating the accuracy of locomotion in rats. *J. Neurosci. Methods* 48 15–25. 10.1016/S0165-0270(05)80003-2 8377518

[B62] Preisig D. F. Kulic L. Krüger M. Wirth F. McAfoose J. Späni C. et al. (2016). High-speed video gait analysis reveals early and characteristic locomotor phenotypes in mouse models of neurodegenerative movement disorders. *Behav. Brain Res.* 311 340–353. 10.1016/J.BBR.2016.04.044 27233823

[B63] Price A. J. Wright F. L. Green J. Balkwill A. Kan S. W. Yang T. Y. O. et al. (2018). Differences in risk factors for 3 types of stroke: UK prospective study and meta-analyses. *Neurology* 90 e298–e306. 10.1212/WNL.0000000000004856 29321237 PMC5798656

[B64] Randomizer, (2024). Available online at: www.randomizer.org (accessed April 1, 2024).

[B65] Ruan J. Yao Y. (2020). Behavioral tests in rodent models of stroke. *Brain Hemorrhages* 1 171–184. 10.1016/J.HEST.2020.09.001 34322665 PMC8315143

[B66] Rust R. Grönnert L. Gantner C. Enzler A. Mulders G. Weber R. Z. et al. (2019). Nogo-A targeted therapy promotes vascular repair and functional recovery following stroke. *Proc. Natl. Acad. Sci. U. S. A.* 116 14270–14279. 10.1073/PNAS.1905309116 31235580 PMC6628809

[B67] Sheppard K. Gardin J. Sabnis G. S. Peer A. Darrell M. Deats S. et al. (2022). Stride-level analysis of mouse open field behavior using deep-learning-based pose estimation. *Cell Rep.* 38:110231. 10.1016/J.CELREP.2021.110231 35021077 PMC8796662

[B68] Shi X. Luo L. Wang J. Shen H. Li Y. Mamtilahun M. et al. (2021). Stroke subtype-dependent synapse elimination by reactive gliosis in mice. *Nat. Commun.* 12:6943. 10.1038/S41467-021-27248-X 34836962 PMC8626497

[B69] Soleman S. Yip P. Leasure J. L. Moon L. (2010). Sustained sensorimotor impairments after endothelin-1 induced focal cerebral ischemia (stroke) in aged rats. *Exp. Neurol.* 222 13–24. 10.1016/J.EXPNEUROL.2009.11.007 19913535 PMC2864515

[B70] Syeara N. Alamri F. F. Jayaraman S. Lee P. Karamyan S. T. Arumugam T. V. et al. (2020). Motor deficit in the mouse ferric chloride-induced distal middle cerebral artery occlusion model of stroke. *Behav. Brain Res.* 380:112418. 10.1016/J.BBR.2019.112418 31812504 PMC6949363

[B71] Syeara N. Bagchi S. Al Shoyaib A. Karamyan S. T. Alamri F. F. Karamyan V. T. (2023). The finer aspects of grid-walking and cylinder tests for experimental stroke recovery studies in mice. *Methods Mol. Biol.* 2616 345–353. 10.1007/978-1-0716-2926-0_23 36715944

[B72] Urbach A. Witte O. W. (2016). Photochemical models of focal brain ischemia. *Neuromethods* 120 69–83. 10.1007/978-1-4939-5620-3_7

[B73] van Nieuwenhuijzen P. S. Parker K. Liao V. Houlton J. Kim H. L. Johnston G. A. R. et al. (2021). Targeting GABAC receptors improves post-stroke motor recovery. *Brain Sci.* 11 1–20. 10.3390/BRAINSCI11030315 33801560 PMC8000079

[B74] von Ziegler L. Sturman O. Bohacek J. (2021). Big behavior: Challenges and opportunities in a new era of deep behavior profiling. *Neuropsychopharmacology* 46 33–44. 10.1038/S41386-020-0751-7 32599604 PMC7688651

[B75] Wang J. Karbasi P. Wang L. Meeks J. P. (2023). A layered, hybrid machine learning analytic workflow for mouse risk assessment behavior. *eNeuro* 10:ENEURO.0335-22.2022. 10.1523/ENEURO.0335-22.2022 36564214 PMC9833056

[B76] Wang R. Tan J. Guo J. Zheng Y. Han Q. So K. F. et al. (2018). Aberrant development and synaptic transmission of cerebellar cortex in a VPA induced mouse autism model. *Front. Cell. Neurosci.* 12:500. 10.3389/FNCEL.2018.00500 30622458 PMC6308145

[B77] Wang Y. Zhang L. Lyu T. Cui L. Zhao S. Wang X. et al. (2024). Association of DNA methylation/demethylation with the functional outcome of stroke in a hyperinflammatory state. *Neural Regen. Res.* 19 2229–2239. 10.4103/1673-5374.392890 38488557 PMC11034580

[B78] Weber R. Z. Mulders G. Kaiser J. Tackenberg C. Rust R. (2022). Deep learning-based behavioral profiling of rodent stroke recovery. *BMC Biol.* 20:232. 10.1186/S12915-022-01434-9 36243716 PMC9571460

[B79] World Health Organization [WHO], (2025). Available online at: https://www.emro.who.int/health-topics/stroke-cerebrovascular-accident/index.html (accessed August 18, 2025).

[B80] Wrench A. Balch-Tomes J. (2022). Beyond the edge: Markerless pose estimation of speech articulators from ultrasound and camera images using DeepLabCut. *Sensors* 22:1133. 10.3390/S22031133 35161879 PMC8838804

[B81] Zahran M. A. Manas-Ojeda A. Navarro-Sánchez M. Castillo-Gómez E. Olucha-Bordonau F. E. (2024). Deep learning-based scoring method of the three-chamber social behaviour test in a mouse model of alcohol intoxication. A comparative analysis of DeepLabCut, commercial automatic tracking and manual scoring. *Heliyon* 10:e36352. 10.1016/J.HELIYON.2024.E36352 39286202 PMC11403434

[B82] Zhan Z. Pan L. Zhu Y. Wang Y. Zhao Q. Liu Y. et al. (2023). Moderate-intensity treadmill exercise promotes mTOR-dependent motor cortical neurotrophic factor expression and functional recovery in a murine model of crush spinal cord injury (SCI). *Mol. Neurobiol.* 60 960–978. 10.1007/S12035-022-03117-6 36385234

[B83] Zhu J. W. Jia W. Q. Zhou H. Li Y. F. Zou M. M. Wang Z. T. et al. (2021). Deficiency of TRIM32 impairs motor function and purkinje cells in mid-aged mice. *Front. Aging Neurosci.* 13:697494. 10.3389/FNAGI.2021.697494 34421574 PMC8377415

[B84] Zörner B. Filli L. Starkey M. L. Gonzenbach R. Kasper H. Röthlisberger M. et al. (2010). Profiling locomotor recovery: Comprehensive quantification of impairments after CNS damage in rodents. *Nat. Methods* 7 701–708. 10.1038/NMETH.1484 20836253

