# Supplementary material for: Development of a deep learning-based grid walking test for assessing post-stroke motor function in mice
Source: Front Behav Neurosci. 2026 Aug 3;20:1873231. doi: 10.3389/fnbeh.2026.1873231 (PMC13478089; doi:10.3389/fnbeh.2026.1873231)

Development of a Deep Learning-Based Grid Walking Test for Assessing Post-Stroke Motor Function in Mice

Supplementary Material

Annotated frames of representative video recordings – back view

Monika Maciag [1], **Vardan T. Karamyan** [1,*]

[1] Department of Foundational Medical Studies, William Beaumont School of Medicine, Oakland University, Rochester, MI 48309, USA

*** Correspondence**:

Vardan T. Karamyan, Pharm.D., Ph.D.

Oakland University William Beaumont School of Medicine

118 Library Drive, Rochester, MI 48309

United States of America

Phone: 248-370-4525

Email: vkaramyan@oakland.edu

Back view annotations (video recording total length 1 second)

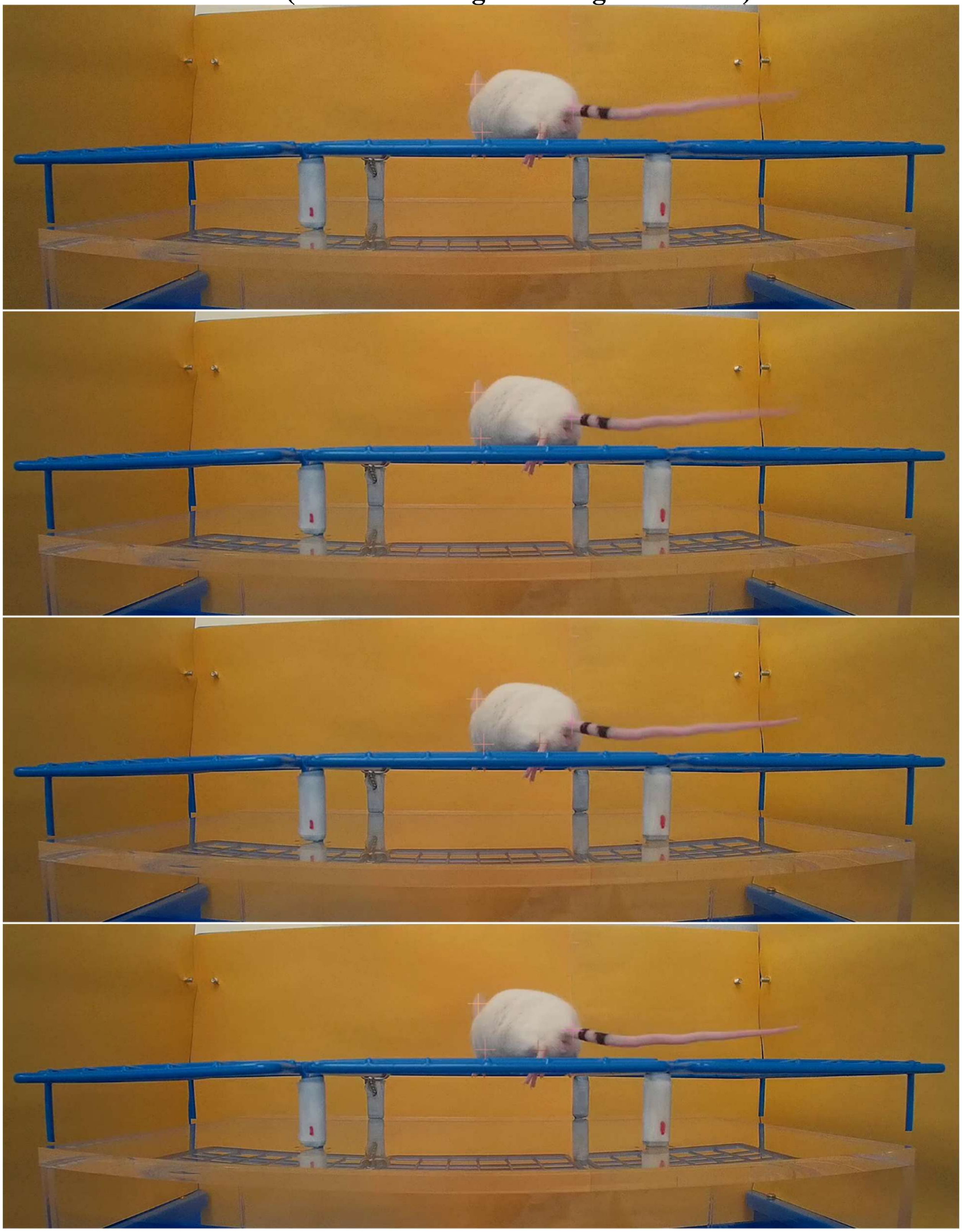

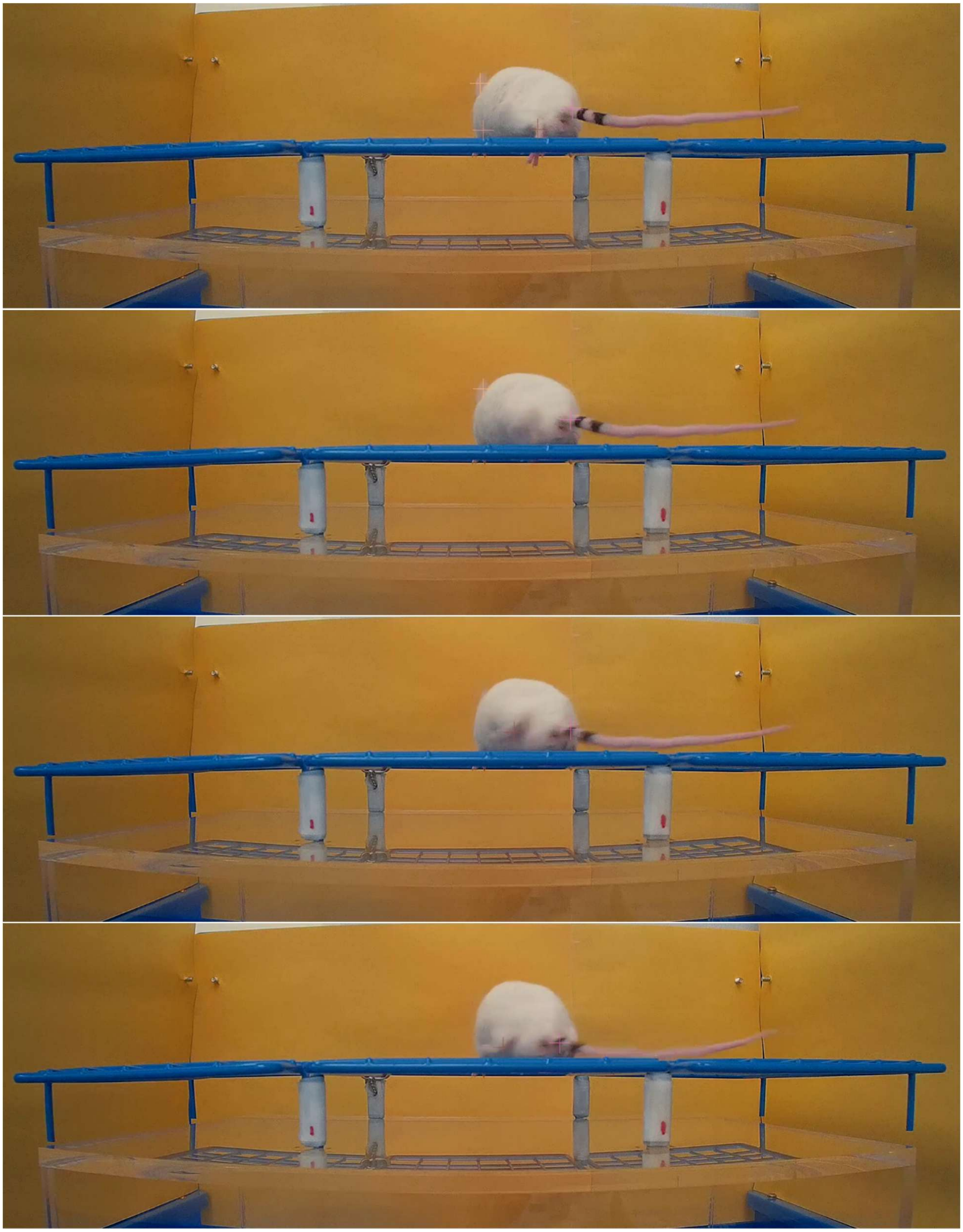

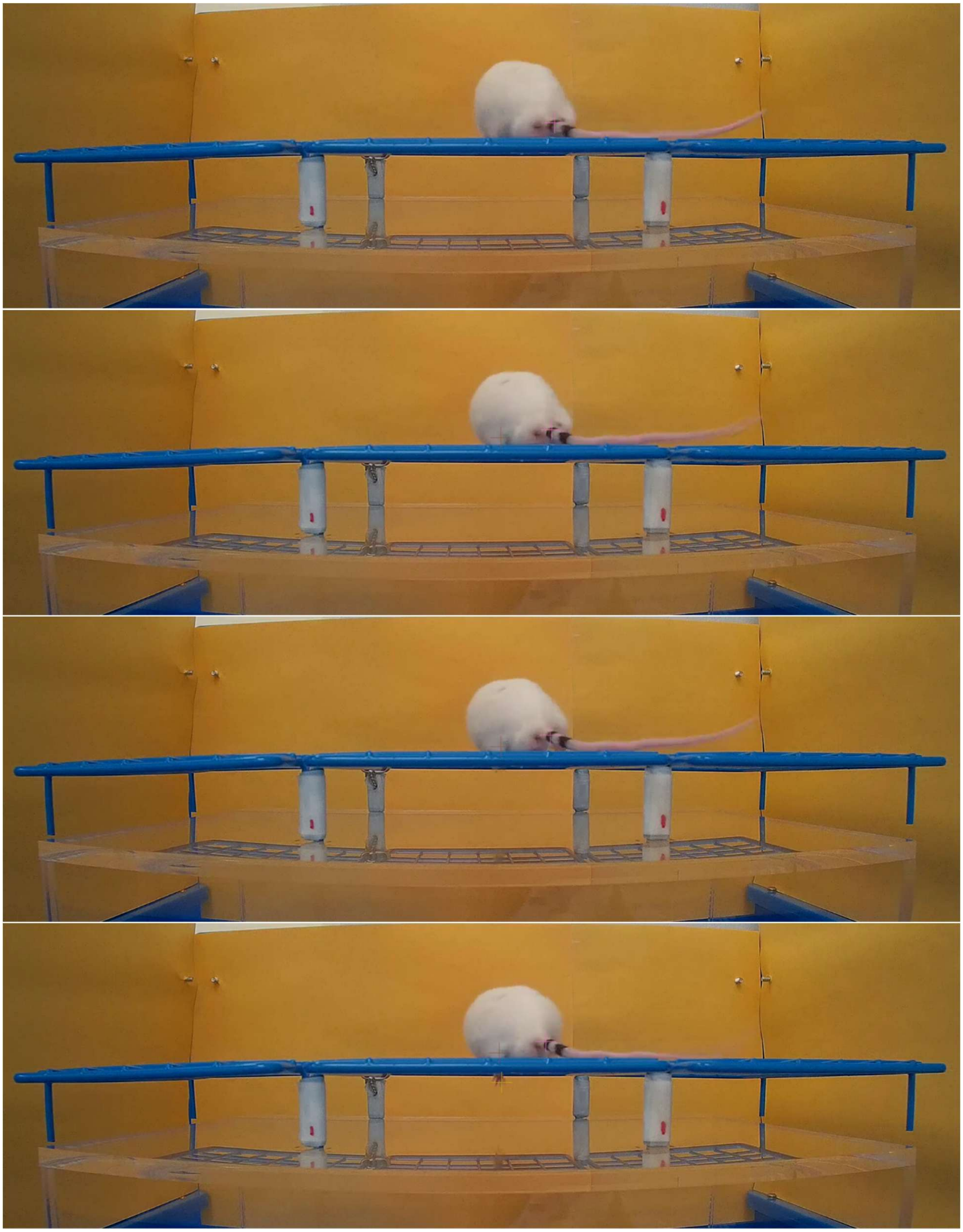

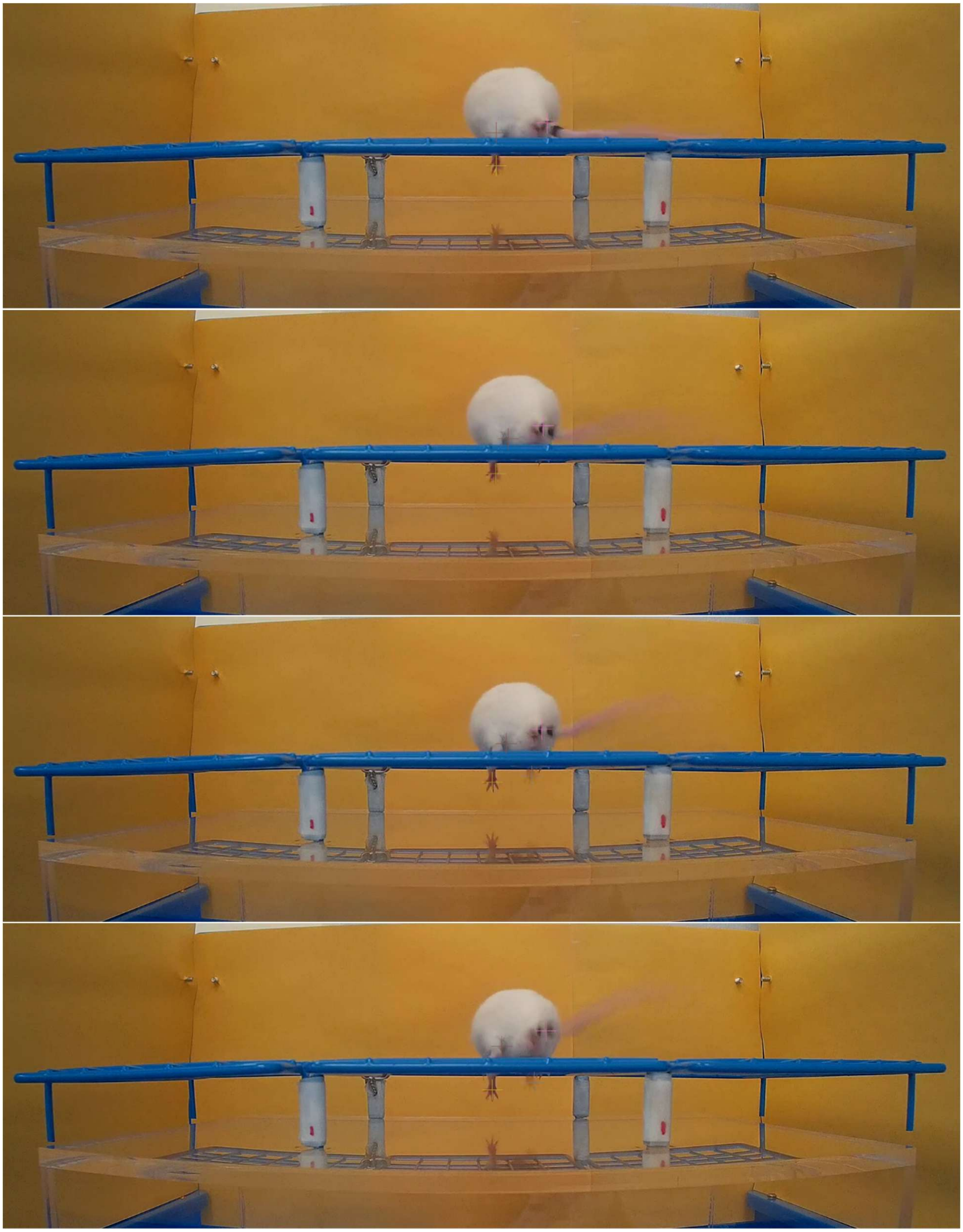

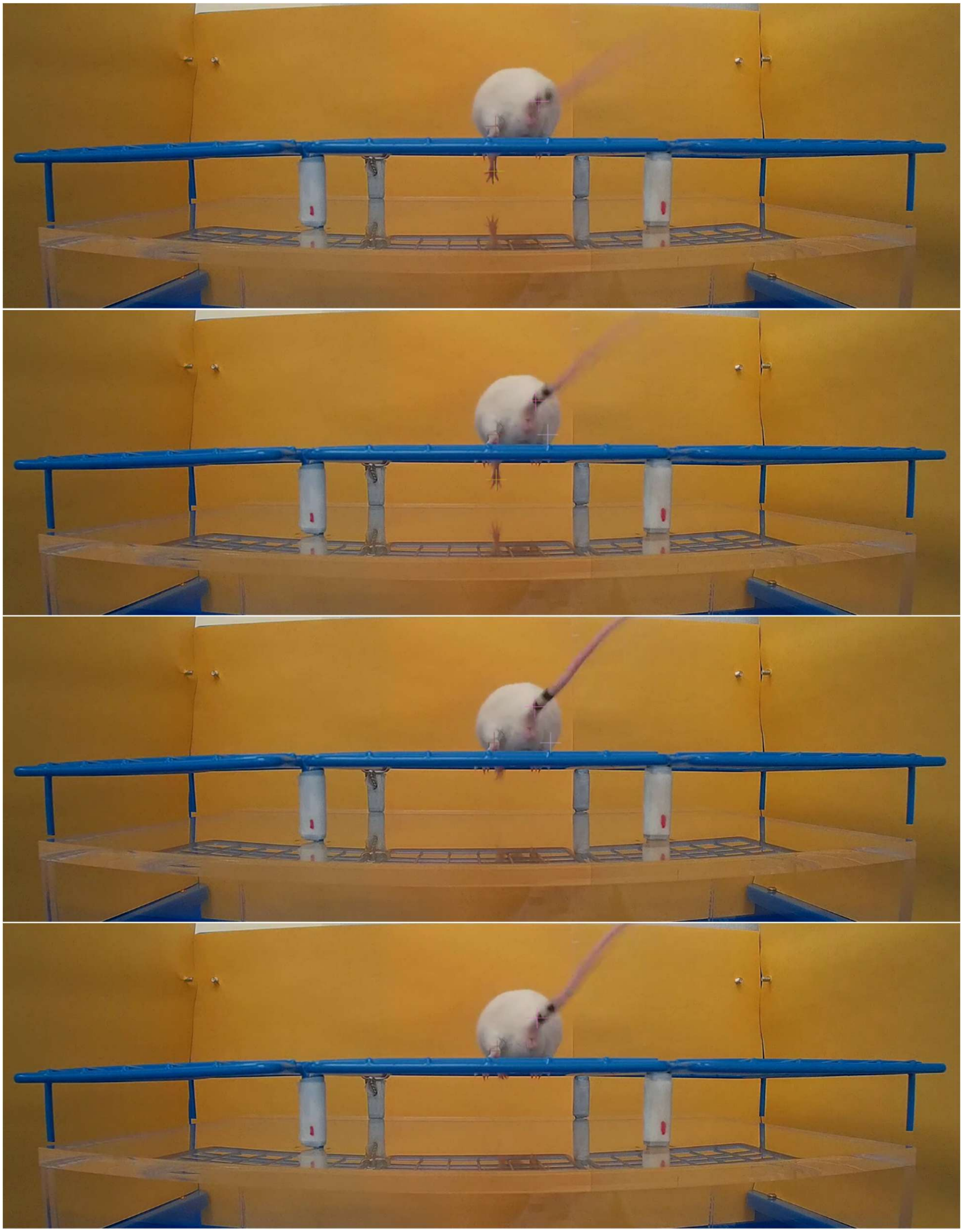

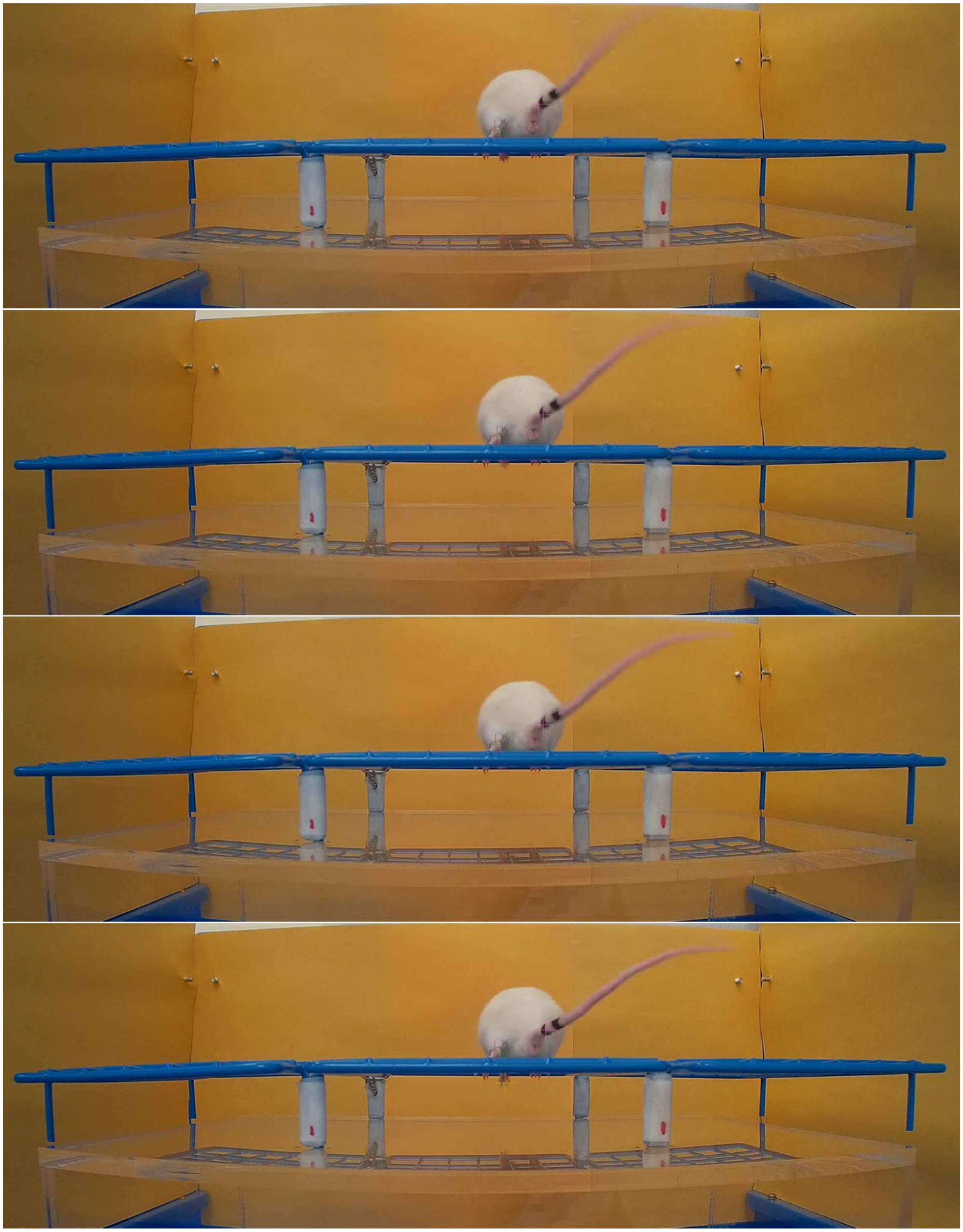

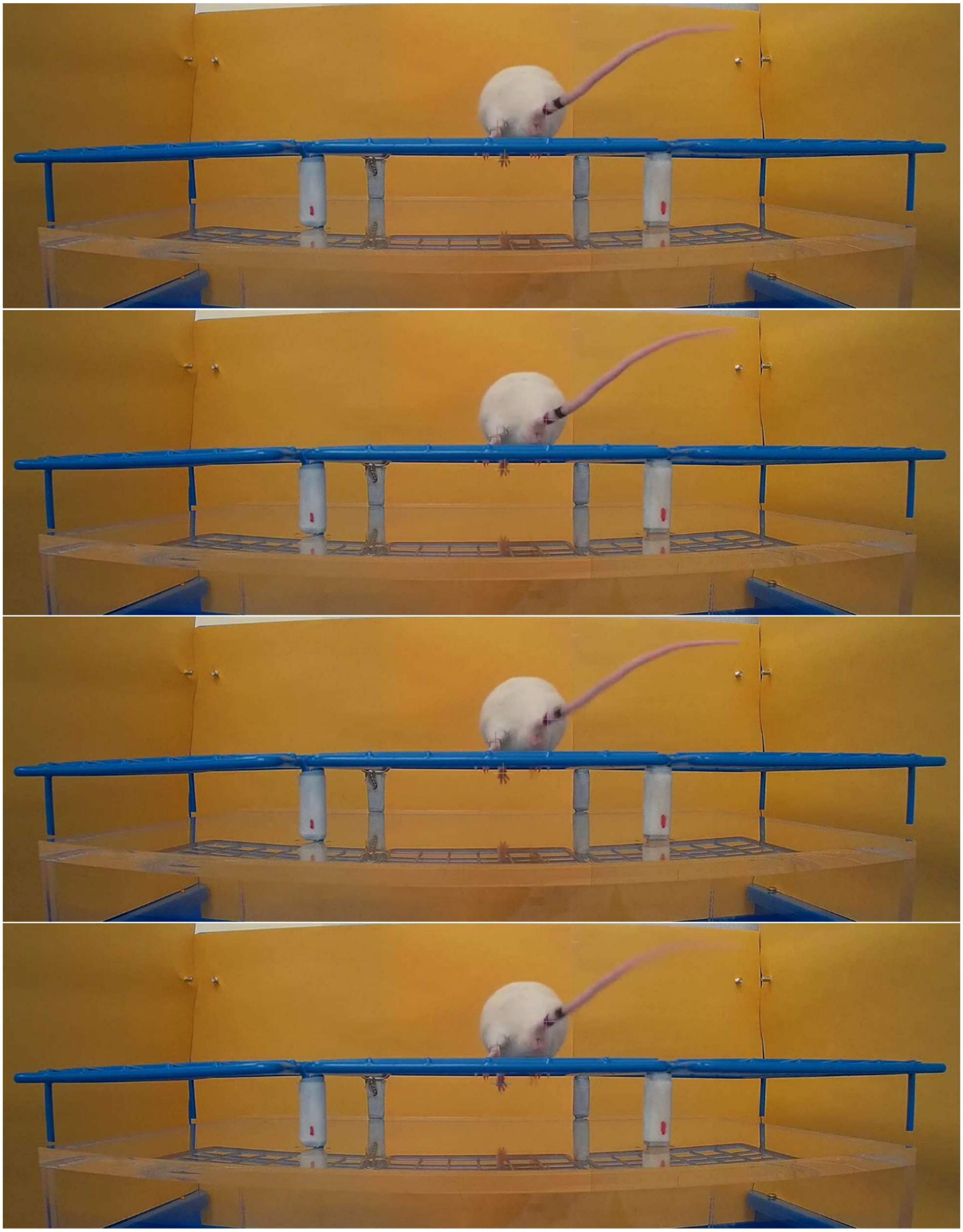

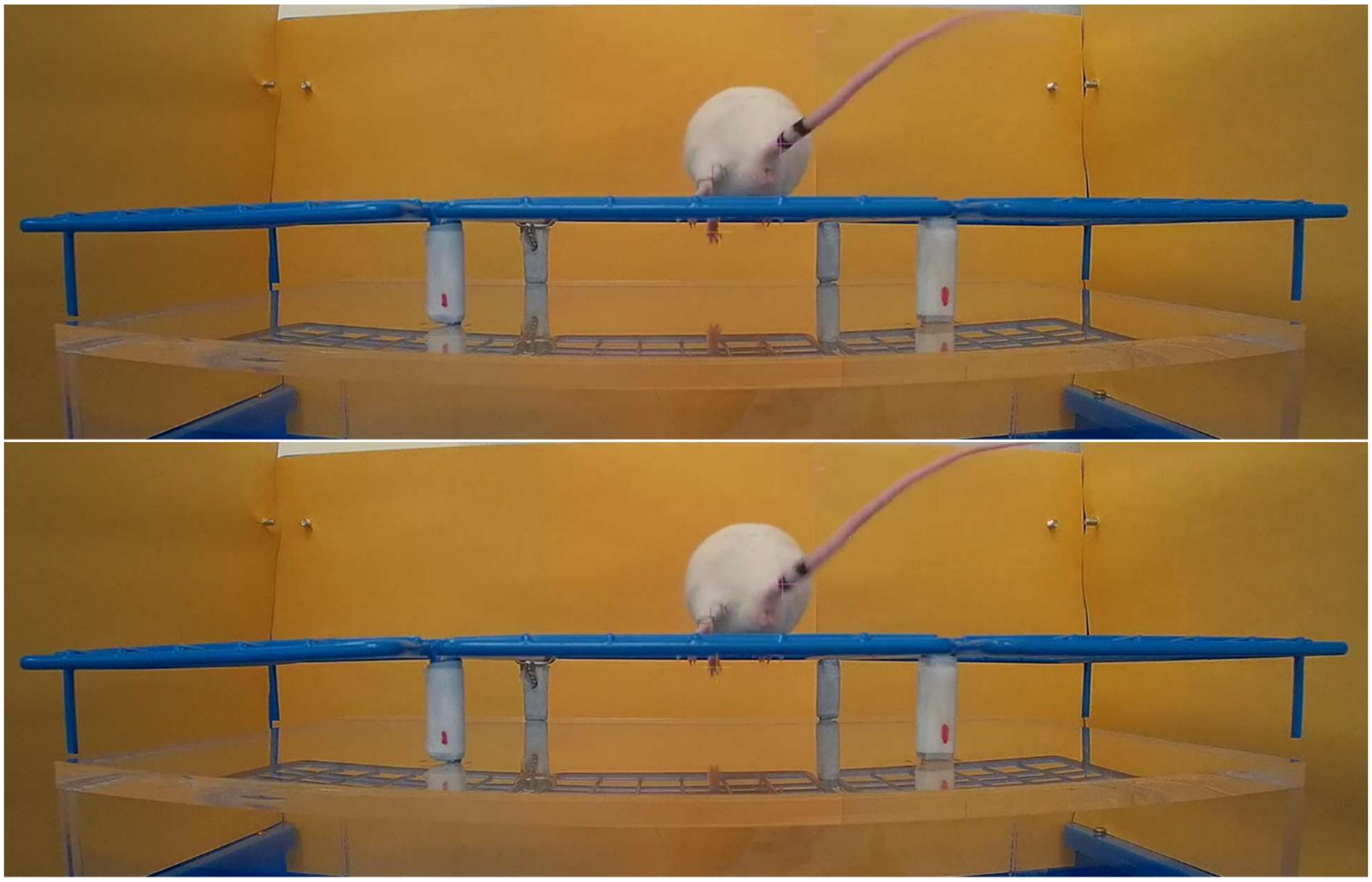

Supplement: Supplementary file 2 [file Data_Sheet_2.pdf]
