# Supplementary material for: Development of a deep learning-based grid walking test for assessing post-stroke motor function in mice
Source: Front Behav Neurosci. 2026 Aug 3;20:1873231. doi: 10.3389/fnbeh.2026.1873231 (PMC13478089; doi:10.3389/fnbeh.2026.1873231)
Supplement: Supplementary file 3 [file Supplementary_file_1.docx]

**Development of a Deep Learning-Based Grid Walking Test for Assessing Post-Stroke Motor Function in Mice**

**Supplementary Material**

**Monika Maciag ^1^, Vardan T. Karamyan ^1, *^**

^1^ Department of Foundational Medical Studies, William Beaumont School of Medicine, Oakland University, Rochester, MI 48309, USA

*** Correspondence**:

Vardan T. Karamyan, Pharm.D., Ph.D.

Oakland University William Beaumont School of Medicine

118 Library Drive, Rochester, MI 48309

United States of America

Phone: 248-370-4525

Email: vkaramyan@oakland.edu

**Table S1 Variability of the stroke-affected forepaw across all testing days based on manual scoring.**

| **STROKE GROUP - AFFECTED PAW** | | |
| --- | --- | --- |
|  | **Mean** | **Standard deviation (SD)** |
| B/line | 7.55 | 2.63 |
| PSD3 | 22.83 | 4.82 |
| PSD7 | 19.22 | 4.12 |
| PSD14 | 15.74 | 3.50 |
| **AVG** | **16.34** | **3.77** |
| **Variability % (SD/mean × 100)** | | **23** |

**Table S2 Mixed-effects model statistics: F-values, partial η² with confidence intervals (95% CI), and Cohen’s f for all effects.**

| **GRID WALKING TEST - MANUAL SCORING** | | | | | | | |
| --- | --- | --- | --- | --- | --- | --- | --- |
|  | Fixed effects (type III) | F (DFn, DFd) | Eta2 (partial) | 95% CI_Lower | 95% CI_Upper | | Cohens_f |
| **Affected Paw** | Time | F (2.375, 42.76) = 31.95 | 0.640 | 0.492 | 0.772 | | 1.333 |
|  | Column Factor | F (1, 18) = 102.4 | 0.850 | 0.739 | 0.931 | | 2.380 |
|  | Time x Column Factor | F (3, 54) = 31.84 | 0.639 | 0.510 | 0.759 | | 1.330 |
| **Un-affected Paw** | Time | F (2.252, 40.54) = 5.571 | 0.236 | 0.074 | 0.477 | | 0.556 |
|  | Column Factor | F (1, 18) = 0.08163 | 0.005 | 0.000 | 0.264 | | 0.071 |
|  | Time x Column Factor | F (3, 54) = 2.837 | 0.136 | 0.037 | 0.352 | | 0.397 |
| **GRID WALKING TEST - DEEPLABCUT SCORING** | | | | | | | |
|  | Fixed effects (type III) | F (DFn, DFd) | Eta2 (partial) | 95% CI_Lower | 95% CI_Upper | | Cohens_f |
| **Affected Paw** | Time | F (2.150, 38.70) = 10.46 | 0.368 | 0.17 | 0.59 | | 0.766 |
|  | Column Factor | F (1, 18) = 22.22 | 0.552 | 0.27 | 0.78 | | 1.106 |
|  | Time x Column Factor | F (3, 54) = 10.43 | 0.367 | 0.21 | 0.56 | | 0.766 |
| **Un-affected Paw** | Time | F (2.295, 41.31) = 3.089 | 0.146 | 0.03 | 0.39 | | 0.420 |
|  | Column Factor | F (1, 18) = 4.946 | 0.216 | 0.01 | 0.56 | | 0.531 |
|  | Time x Column Factor | F (3, 54) = 3.171 | 0.150 | 0.04 | 0.37 | | 0.420 |
| *** Un-affected Paw** | Time | F (+infinity, +infinity) = |  |  |  | |  |
|  | Column Factor | F (1, 72) = |  |  |  | |  |
|  | Time x Column Factor | F (3, 72) = |  |  |  | |  |
| **AUTOMATED LADDER TEST- 15 RUNS** | | | | | | | |
|  | Fixed effects (type III) | F (DFn, DFd) | Eta2 (partial) | 95% CI_Lower | 95% CI_Upper | | Cohens_f |
| **Affected Paw** | Time | F (2.461, 44.31) = 0.7337 | 0.039 | 0.005 | 0.247 | | 0.201 |
|  | Column Factor | F (1, 18) = 7.102e-007 | 3.95E-08 | 5.61E-05 | 0.249 | | 0.000 |
|  | Time x Column Factor | F (3, 54) = 0.7297 | 0.039 | 0.008 | 0.230 | | 0.201 |
| **Un-affected Paw** | Time | F (2.326, 41.86) = 2.650 | 0.128 | 0.022 | 0.368 | | 0.383 |
|  | Column Factor | F (1, 18) = 0.03029 | 0.002 | 5.78E-05 | 0.255 | | 0.045 |
|  | Time x Column Factor | F (3, 54) = 1.676 | 0.085 | 0.018 | 0.293 | | 0.305 |
| **AUTOMATED LADDER TEST- INITIAL 5 RUNS** | | | | | | | |
|  | Fixed effects (type III) | F (DFn, DFd) | Eta2 (partial) | 95% CI_Lower | 95% CI_Upper | | Cohens_f |
| **Affected Paw** | Time | F (2.606, 46.92) = 2.300 | 0.113 | 0.022 | 0.339 | | 0.357 |
|  | Column Factor | F (1, 18) = 0.2076 | 0.011 | 6.90E-05 | 0.284 | | 0.105 |
|  | Time x Column Factor | F (3, 54) = 1.277 | 0.066 | 0.013 | 0.269 | | 0.266 |
| **Un-affected Paw** | Time | F (2.773, 49.91) = 5.235 | 0.225 | 0.083 | 0.446 | | 0.539 |
|  | Column Factor | F (1, 18) = 0.6629 | 0.036 | 1.09E-04 | 0.341 | | 0.193 |
|  | Time x Column Factor | F (3, 54) = 3.001 | 0.143 | 0.040 | 0.359 | | 0.408 |
| **AUTOMATED LADDER TEST- LAST 5 RUNS** | | | | | | | |
|  | Fixed effects (type III) | F (DFn, DFd) | Eta2 (partial) | 95% CI_Lower | 95% CI_Upper | | Cohens_f |
| **Affected Paw** | Time | F (2.376, 42.76) = 2.833 | 0.136 | 0.026 | 0.374 | | 0.397 |
|  | Column Factor | F (1, 18) = 0.2490 | 0.014 | 7.20E-05 | 0.290 | | 0.119 |
|  | Time x Column Factor | F (3, 54) = 0.5563 | 0.030 | 0.007 | 0.216 | | 0.176 |
| **Un-affected Paw** | Time | F (2.323, 41.82) = 0.1948 | 0.011 | 0.003 | 0.200 | | 0.105 |
|  | Column Factor | F (1, 18) = 0.6386 | 0.034 | 1.06E-04 | 0.338 | | 0.188 |
|  | Time x Column Factor | F (3, 54) = 0.8769 | 0.046 | 0.009 | 0.242 | | 0.220 |
| **AUTOMATED LADDER TEST - Steps/s** | | | | | | | |
|  | Fixed effects (type III) | F (DFn, DFd) | Eta2 (partial) | 95% CI_Lower | 95% CI_Upper | Cohens_f | |
| **Affected Paw** | Time | F (2.281, 41.06) = 0.8783 | 0.047 | 0.005 | 0.265 | 0.222 | |
|  | Column Factor | F (1, 18) = 0.1858 | 0.010 | 6.75E-05 | 0.281 | 0.101 | |
|  | Time x Column Factor | F (3, 54) = 0.8240 | 0.044 | 0.009 | 0.238 | 0.215 | |
| **Un-affected Paw** | Time | F (2.245, 40.41) = 0.5385 | 0.029 | 0.003 | 0.238 | 0.173 | |
|  | Column Factor | F (1, 18) = 0.1860 | 0.010 | 6.76E-05 | 0.281 | 0.101 | |
|  | Time x Column Factor | F (3, 54) = 0.5470 | 0.029 | 0.007 | 0.215 | 0.173 | |
| **AUTOMATED LADDER TEST - Steps/s** | | | | | | | |
|  | Fixed effects (type III) | F (DFn, DFd) | Eta2 (partial) | 95% CI_Lower | 95% CI_Upper | Cohens_f | |
| **Sham** | Time | F (2.361, 42.49) = 0.7924 | 0.042 | 0.005 | 0.256 | 0.209 | |
|  | Column Factor | F (1, 18) = 0.0001163 | 6.46E-06 | 5.61E-05 | 0.249 | 0.003 | |
|  | Time x Column Factor | F (3, 54) = 0.01876 | 0.001 | 0.004 | 0.160 | 0.032 | |
| **Stroke** | Time | F (1.693, 30.48) = 1.935 | 0.097 | 0.005 | 0.366 | 0.328 | |
|  | Column Factor | F (1, 18) = 0.0003270 | 1.82E-05 | 5.61E-05 | 0.249 | 0.004 | |
|  | Time x Column Factor | F (3, 54) = 0.07089 | 0.004 | 0.004 | 0.167 | 0.063 | |
| **GRID WALKING TEST - MANUAL-DEEPLABCUT SCORING - Steps/s** | | | | | | | |
|  | Fixed effects (type III) | F (DFn, DFd) | Eta2 (partial) | 95% CI_Lower | 95% CI_Upper | Cohens_f | |
| **Sham** | Time | F (2.475, 44.55) = 10.10 | 0.359 | 0.181 | 0.570 | 0.748 | |
|  | Column Factor | F (1, 18) = 1.121 | 0.059 | 1.72E-04 | 0.382 | 0.250 | |
|  | Time x Column Factor | F (3, 54) = 1.489 | 0.076 | 0.015 | 0.282 | 0.287 | |
| **Stroke** | Time | F (2.569, 46.24) = 39.60 | 0.688 | 0.561 | 0.799 | 1.485 | |
|  | Column Factor | F (1, 18) = 0.06932 | 0.004 | 6.01E-05 | 0.262 | 0.063 | |
|  | Time x Column Factor | F (3, 54) = 0.08916 | 0.005 | 0.004 | 0.169 | 0.071 | |

* Unaffected forepaw, cut-off 0.7: only 0 values observed for both sham and stroke groups across all days.

**Figure S1 The automated ladder test fails to detect motor impairments in post-stroke mice.** The commercially available automated ladder test (BIOSEB) did not reveal significant increases in missteps for either the affected or unaffected forepaws on post-stroke days 3, 7, or 14 (PSD3, PSD7, PSD14) when analyzing (**A** and **B**) the first 5 trials and (**C** and **D**) the last 5 trials. Step frequency analysis of the left and right forelimbs showed no significant differences between stroke and sham groups for either the (**E**) affected or (**F**) unaffected paws, nor (**G**) sham or (**H**) stroke, with animals consistently averaging approximately two steps per second across all time points. Data were analyzed using a repeated measures mixed-effects model followed by Tukey's post-hoc test. *P*-values < 0.05 were considered statistically significant. The bold, solid and dashed lines represent the mean data ± standard error of the mean (SEM), n = 10/group. The faint lines show the data for individual animals in each experimental group.

**Figure S2 DeepLabCut training accuracy and validation of misstep and step detection consistency.** (**A**) Root mean square errors (RMSEs) across anatomical landmarks in the training dataset ranged from 0.06 to 0.12 cm, while misstep labeling errors ranged from 0.10 to 0.33 cm, indicating consistent sub-centimeter precision. The slightly higher error for right forepaw missteps may be attributed to a smaller number of annotated frames used during model training for that limb. (**B**) Using a uniform 0.7 threshold across paws artificially suppresses misstep detection on the unaffected side, showing zero missteps. The number of steps detected using the DLC-based model was compared with manually scored data. DLC-derived step counts were nearly identical to manual annotations, with no significant differences observed in either the (**C**) sham or (**D**) stroke groups. Mice performed approximately one step per second across conditions. Data were analyzed using a repeated measures mixed-effects model followed by Tukey’s post hoc test. *P*-values < 0.05 were considered statistically significant. The bold, solid and dashed lines represent the mean data ± standard error of the mean (SEM), n = 10/group. The faint lines show the data for individual animals in each experimental group.

**Table S3 Root Mean Square Error (RMSE) values across five independent random shuffles of training and test frames.**

| **DEEPLABCUT EVALUATION** | | | | |
| --- | --- | --- | --- | --- |
| Shuffle Number | Train RMSE (px) | Test RMSE (px) | Train Error (cm) | Test Error (cm) |
| 1 | 6.65 | 27.94 | 0.095 | 0.399 |
| 2 | 5.19 | 7.28 | 0.074 | 0.104 |
| 3 | 4.59 | 8.61 | 0.066 | 0.123 |
| 4 | 4.42 | 6.74 | 0.063 | 0.096 |
| 5 | 6.34 | 7.60 | 0.091 | 0.109 |

**Figure S3 Rostro-caudal infarct position influences motor impairment.** The rostro-caudal infarct position was associated with motor outcome, with more rostral lesions predicting fewer missteps in (**A**) the manual dataset and showing a similar, though non-significant, trend in (**B**) the DLC-based analysis. Data were analyzed using Pearson correlation. *P*-values < 0.05 were considered statistically significant. Data are shown as a mean ± standard error of the mean (SEM), *n* = 6/group.

**Figure S4. Histogram showing the distribution of differences in misstep percentages between manual and DLC-based scoring.** The distribution demonstrates a mean discrepancy of approximately 3 percentage points between the two scoring approaches (n=160/group).
